# Covalent organic framework-based heterojunction photocatalysts

**DOI:** 10.1093/nsr/nwaf462

**Published:** 2025-10-29

**Authors:** Jingzhao Cheng, Wang Wang, Shaowen Cao, Yaqian Lan

**Affiliations:** State Key Laboratory of Advanced Technology for Materials Synthesis and Processing, School of Material Science and Engineering, Wuhan University of Technology, Wuhan 430070, China; Hubei Technology Innovation Center for Advanced Composites, Wuhan University of Technology, Wuhan 430070, China; State Key Laboratory of Advanced Technology for Materials Synthesis and Processing, School of Material Science and Engineering, Wuhan University of Technology, Wuhan 430070, China; Hubei Technology Innovation Center for Advanced Composites, Wuhan University of Technology, Wuhan 430070, China; State Key Laboratory of Advanced Technology for Materials Synthesis and Processing, School of Material Science and Engineering, Wuhan University of Technology, Wuhan 430070, China; Hubei Technology Innovation Center for Advanced Composites, Wuhan University of Technology, Wuhan 430070, China; Guangdong Provincial Key Laboratory of Carbon Dioxide Resource Utilization, School of Chemistry, South China Normal University, Guangzhou 510006, China

**Keywords:** covalent organic framework, heterojunction, photocatalysis, solar-to-energy conversion

## Abstract

Photocatalysis, as a sustainable, green and low-cost technology that converts solar energy into chemical energy, holds great potential to address the issues of environmental pollution and energy shortages. However, the rapid charge recombination in semiconductors limits their potential for achieving high photocatalytic activity. Heterojunctions, including inorganic-inorganic, organic-inorganic, and organic-organic heterojunctions, have proven to be an effective strategy for inhibiting charge recombination. By virtue of the high crystallinity, tunable structure and accessible pore systems of covalent organic frameworks (COFs), COF-based heterojunction photocatalysts have shown appealing prospects for promoting solar-to-energy conversion by improving charge separation, enhancing light-harvesting ability and facilitating photoredox reactions. In this review, the state-of-the-art progress in COF-based heterojunction photocatalysts including their mechanism, classification, synthetic strategies and applications are summarized. In particular, the synergistic effect and charge transfer mechanism as well as the structure–activity relationships of COF-based heterojunctions are thoroughly reviewed. Finally, the challenges and outlooks of COF-based heterojunction photocatalysts are also discussed. It is believed that this review can stimulate inspiration for the future development of efficient COF-based heterojunction photocatalysts.

## Teaser

This review summarizes the state-of-the-art progress in COF-based heterojunction photocatalysts, covering their mechanism, classification, synthesis and applications, offering valuable insights for the future development of efficient COF-based heterojunction photocatalysts.

## INTRODUCTION

**Figure 1.**
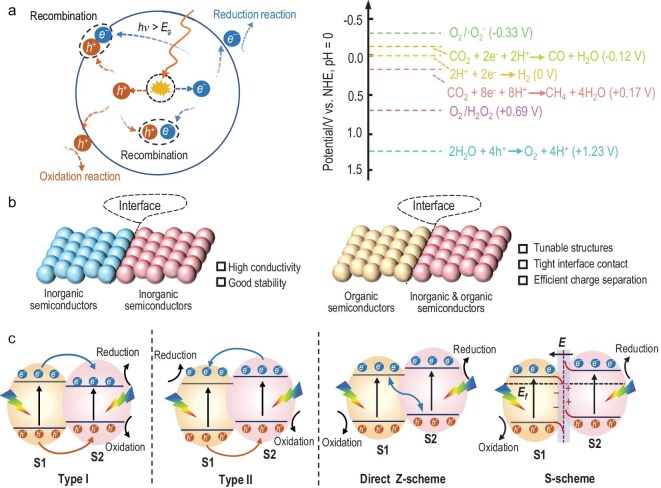
(a) Schematic illustration of the basic principle and energy diagrams of photoredox reaction. Schematic illustration of heterojunction types according to (b) the types of constituent materials and (c) the distinct configurations of band alignment.

The extensive use of fossil fuels has led to two pressing challenges: the energy crisis and environmental pollution [1–3]. With the increasing depletion of non-renewable resources, the pursuit of clean renewable energy and addressing environmental pollution have emerged as global priorities [4–6]. Photocatalysis, inspired by natural photosynthesis, has emerged as a field in response to these challenges. This technology holds immense potential for a wide range of energy and environmental applications by harnessing clean and sustainable solar energy. In semiconductor photocatalytic systems, photogenerated electrons and holes have sufficient redox potential to drive the redox reactions required for fuel generation (Fig. 1a) [7–10]. Various traditional semiconductors have been developed in the field of photocatalysis, including metal oxides (MOs, such as WO_3_ [11], TiO_2_ [12], Cu_2_O [13]) and metal sulfides (MSs, such as CdS [14–16], ZnS [17], ZnInS_4_ [18]), but they often suffer from limitations such as low solar energy utilization, low surface areas and rapid charge recombination [19]. Therefore, researchers have turned to the development of heterojunction photocatalysts, which combine materials with complementary properties to enhance overall photocatalytic performance [20].

Heterojunctions, where two or more materials are combined to form an interface that facilitates charge separation, have proven to be an effective strategy for improving photocatalytic efficiency [21,22]. As shown in Fig. 1b, these heterojunctions can be classified according to the types of constituent materials: inorganic-inorganic, organic-inorganic, and organic-organic. The highly ordered lattice structure in inorganic-inorganic semiconductor heterojunctions endows them with high electron mobility and good stability, but it is difficult to achieve flexible assembly and modification. Compared to inorganic-inorganic heterojunctions, organic-inorganic or organic-organic heterojunctions offer greater flexibility [23]. Specifically, the diversity in monomers and synthesis methods enables organic semiconductors to have tunable molecular structures. Upon forming heterojunctions, organic molecules containing heteroatoms such as N, O and S can interact with metal ions at the interface. Usually, the heteroatoms have lone pairs of electrons, which can act as Lewis bases. These lone pairs of electrons can interact with the vacant orbitals of metal ions, leading to the formation of coordinated bridge structures at the interface and enhancing the contact between the organic semiconductors and inorganic semiconductors. This strong interface interaction further promotes efficient charge separation. Furthermore, the manipulation of the electronic properties and molecular architecture of organic semiconductors allows for the fine-tuning of band alignments at the heterojunction interface, optimizing charge transfer and improving photocatalytic performance [24]. According to the distinct configurations of band alignment and interaction between the constituent materials, the heterojunctions can be classified into three types (Fig. 1c). The type I heterojunction involves two semiconductors (semiconductor S2 features a higher conduction band (CB) level than semiconductor S1) with a straddling band structure. Under light irradiation, the electron and hole from S2 are transferred directly to the CB and valence band (VB) of S1, respectively, which confines the photogenerated charge carriers to S2 [25]. The type II heterojunction is formed by a staggered gap between S1 and S2, where the electron in S2 moves to S1, and the hole in S1 moves to S2. This transfer process enhances spatial charge separation and prolongs carrier lifetime [26–28]. Direct Z-scheme or S-scheme heterojunctions enable the retention of photogenerated electrons and holes with the strongest redox abilities, effectively enhancing charge separation and transfer efficiency. Compared to the direct Z-scheme heterojunction, the S-scheme heterojunction offers a more rational interpretation of the charge transfer mechanism based on the interface electric field. For S-scheme heterojunctions, when semiconductors S1 and S2 come into contact, the Fermi level (*E*_f_) aligns through electron flow, leading to the band bending at the interface and forming a built-in electric field directed toward S1. This electric field promotes charge migration by driving photogenerated electrons from the CB of S2 to recombine with holes in the VB of S1 at the interface. As a result, the remaining electrons in the CB of S1 and holes in the VB of S2 are spatially separated, suppressing recombination and enabling efficient photoredox reactions.

**Figure 2.**
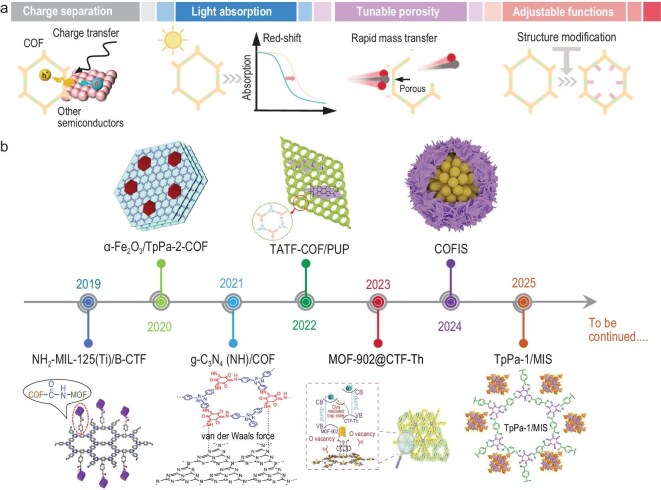
(a) The advantages of COF-based heterojunction photocatalysts. (b) Timeline for the development of representative COF-based heterojunction photocatalysts. From left: NH_2_-MIL-125(Ti)B-CTF [62]; Copyright 2019, Elsevier. *α*-Fe_2_O_3_/TpPa-2-COF [63]; Copyright 2020, The Royal Society of Chemistry. g-C_3_N_4_ (NH)/COF [64]; Copyright 2022, Elsevier. TATF-COF/PUP [65]; Copyright 2022, Elsevier. MOF-902@CTF-Th [66]; Copyright 2023, Wiley-VCH. COFIS [67]; Copyright 2024, Wiley-VCH. TpPa-1/MIS [68]. Copyright 2025, Wiley-VCH.

Covalent organic frameworks (COFs) have become a highly promising class of organic materials for the construction of heterojunctions due to their unique structural and functional properties [29–33]. COFs are crystalline porous materials constructed by linking multi-functional organic building blocks through covalent bonds, offering highly ordered structures with precise molecular arrangements, synthetic versatility, and solid-state properties. These properties provide COFs with tremendous potential in the field of heterogeneous photocatalysts [34–36]. It can be expected that the construction of COF-based heterojunctions has great potential in promoting efficient charge separation and improving solar-to-chemical conversion (Fig. 2a) [37]. For wide band gap photocatalysts, limited visible light absorption hinders their photocatalytic efficiency. By combining them with COFs, light absorption can be extended into the visible-light range. Additionally, the structural multi-functions of COFs allow precise control over pore size, functional groups, and electronic properties, making them adaptable for various photocatalytic applications. Their porous structure also allows for effective mass transfer of reactants to the surface of the photocatalyst, ensuring abundant reactants for the photocatalytic reactions, thereby improving overall photocatalytic efficiency. Moreover, the versatile molecular structures of COF-based heterojunctions enable facile structure modifications, allowing for the precise tuning of their electronic properties and photocatalytic performance, with enhanced flexibility achieved through functionalization with complementary materials. Based on these advantages, COF-based heterojunctions are expected to play a critical role in the development of efficient photocatalytic systems, contributing to enhanced charge separation and improved solar-to-chemical conversion [38]. The construction of heterojunctions has great potential in promoting efficient charge separation and improving photocatalytic performance [39,40]. A comprehensive understanding of the charge transfer process in COF-based heterojunctions is crucial for tuning band gaps and enhancing the efficiency of charge separation [41].

In this review, the progress on COF-based heterojunction photocatalysts is covered, including charge transfer mechanism, classification, synthetic strategies and applications in the fields of pollutant photodegradation, H_2_ evolution, CO_2_ reduction, H_2_O_2_ production and organic synthesis. The synergistic effect of COFs and other semiconductor materials is highlighted. Besides, this review also points out the inherent challenges and outlooks of COF-based heterojunctions. It is hoped that this review can deepen the understanding of COF-based heterojunction photocatalysts and stimulate inspiration for the development and design of high-activity photocatalysts in the future.

## THE CLASSIFICATION FOR COF-BASED HETEROJUNCTION PHOTOCATALYSTS

**Figure 3.**
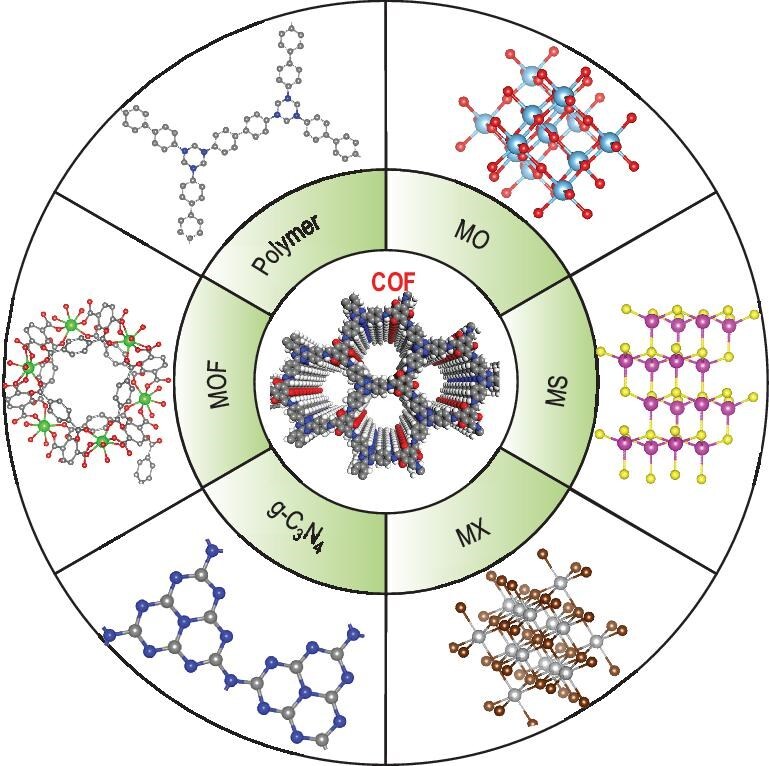
Summary of materials used to construct COF-based S-scheme heterojunctions.

Since the first report of COFs in 2005 [42], numerous scientists have advanced the field of COF-based heterostructures, leading to a rapid increase in publications in recent years (Fig. 2b). The classification of COF-based heterojunctions plays a crucial role in understanding their diverse applications and tailored functionalities. From the perspective of different substrate chemical compositions, COF-based heterostructure photocatalysts in this review are mainly divided into six categories (Fig. 3): (1) MO/COF [43]; (2) MS/COF [44–46]; (3) Metal halides/COF (labeled as MX/COF) [47–49]; (4) g-C_3_N_4_/COF [50–53]; (5) Metal-organic framework (MOF)/COF [54–56]; (6) Polymer/COF [57,58]; and others (metal phosphate, layered double hydroxide and carbon nanomaterials)/COF [59–61].

### MO/COF

MO/COF heterojunction photocatalysts integrate the excellent photocatalytic activity of MO with the structural tunability and surface functionalization capabilities of COFs. Metal oxides such as TiO_2_ [69], ZnO [70] and CeO_2_ [71] offer low cost, good stability, and efficient photogenerated charge separation. However, common MO materials generally suffer from issues including weak reduction ability and easy recombination of photogenerated charge carriers, which restricts their photocatalytic efficiency. COFs, with highly controllable structures, allow precise tuning of pore structure and surface chemistry through targeted design and synthesis. For instance, Yang *et al*. [72] reported an S-scheme TiO_2_@BTTA compound. Compared with pure TiO_2_ and BTTA, the TB-6 hybrid exhibited a smaller semicircle radius in electrochemical impedance spectroscopy Nyquist plots and higher photocurrent intensity, indicating that the S-scheme heterostructure reduced electron transfer resistance and enhanced charge carrier migration. The strongest DMPO-^•^O_2_^−^ signal was observed on composite material, demonstrating that TiO_2_@BTTA possesses a potent reducing ability. Under light irradiation, the electron transfer of TiO_2_@BTTA followed the S-scheme mechanism: photogenerated electrons of TiO_2_ transferred to and combined with the holes in BTTA, retaining electrons and holes with strong redox capacity. Electrons reduced O_2_ to H_2_O_2_ by two steps, while the hole activated the *α*-C–H of furfuryl alcohol (FA) to produce ^•^C_5_H_5_O_2_. The final product was formed via the reaction of ^•^C_5_H_5_O_2_ with ^•^OH, followed by dehydrogenation. The optimized TB-6 achieved an H_2_O_2_ yield of 740 μmol L^−1^ h^−1^ and 96% FA oxidation. Notably, the TiO_2_/BTTA composite exhibited a large interfacial area and short carrier migration paths, as BTTA grew *in situ* on the TiO_2_ surface, forming a core-shell hybrid structure. Additionally, the porous and ultrathin BTTA layer endowed the heterostructure with abundant active sites and enhanced light absorption. Researchers have further demonstrated that the transfer of charge carriers across heterostructure interfaces strongly depends on the exposed surface of the MO. Qu and co-workers studied the influence of surface characteristics on TiO_2_/Tp-Tta COF S-scheme heterojunctions [73]. They found that the electron-rich properties and lower CB level of 101-TiO_2_ promoted efficient recombination of electrons in the CB of TiO_2_ with holes in the VB of COF, enhancing charge transfer efficiency. The CO_2_ reduction rate of the composite reached 11.6 μmol h^−1^, which is 14.5 and 4.6 times higher than that of the original 101-TiO_2_ and T-001/COF heterostructure, respectively. Recently, Fe-porphyrin and terephthalaldehyde were anchored on the surface of amino-functionalized CeO_2_ to form a COF-366-Fe@CeO_2_ core-shell structure [71]. The Z-scheme charge transfer mechanism at COF-366-Fe@CeO_2_ promotes photogenerated electron separation and migration, leveraging the efficient reduction capability of COF-366-Fe and the strong oxidation capacity of CeO_2_. In these hybrids, Fe(III) is reduced to Fe(II) due to the anchoring effect in the metal porphyrin, providing an additional way for electrons to migrate rapidly to the dispersed Fe centers, thereby improving the capture and conversion efficiency of CO_2_. Fang *et al*. [74] developed a stable COF-based S-scheme heterojunction through solvothermal synthesis of WO_3_ and metal-covalent organic frameworks (M-COFs) for photocatalytic CO_2_ reduction. Under visible-light irradiation in a gas–solid system with pure CO_2_ and water vapor, the optimized WO_3_/THFB-COF-Zn (3:7) achieved a CO production rate of 54.1 µmol g^−1^ h^−1^ without photosensitizers or sacrificial agents, which is ∼7 times higher than that of pristine THFB-COF-Zn (8 µmol g^−1^ h^−1^). The photocatalytic CO_2_ reduction is coupled with H_2_O oxidation, yielding a CO/O_2_ ratio of ∼2:1. Stability tests over ten cycles confirmed robust performance with retained structural integrity.

### MS/COF

Constructing MS/COF heterojunctions is an effective strategy to mitigate photocorrosion in MS and improve overall photocatalytic efficiency. MS, such as MoS_2_ [75], ZnIn_2_S_4_ [76], ZnCdS [77] and CdS [78] exhibit excellent electronic properties and good light absorption ability, making them ideal candidates for photocatalysis. However, the lattice S^2−^ in these materials is easily oxidized by photogenerated holes when exposed to light for a prolonged period of time, resulting in reduced catalyst stability and deactivation [79,80]. The tuning band structure of COFs can stabilize MS to prevent photocorrosion. Zhang’s group prepared a Z-scheme heterojunction photocatalyst using ZnIn_2_S_4_ as a reduced semiconductor and TPA-1-COF as an oxidized semiconductor [76]. The density functional theory (DFT) calculation showed that the work function (*W*_f_) of ZnIn_2_S_4_ and TPA-1-COF is 4.63 and 4.82 eV, respectively. Therefore, electrons flow from ZnIn_2_S_4_ to TPA-1-COF after contact, resulting in the band bending and formation of an internal electric field at the interface directed toward the TPA-1-COF. The electrons accumulated in the CB of TPA-1-COF migrate to the VB of ZnIn_2_S_4_ driven by the built-in electric field under light irradiation. This directional motion not only inhibits the electron-hole recombination but also maximizes the reduction ability of the composite, thereby improving its photocatalytic hydrogen production activity. Besides, the photocorrosion of ZnIn_2_S_4_ was inhibited because the photogenerated holes in ZnIn_2_S_4_ were consumed by the electrons on PA-1-COF, thus improving the stability of ZnIn_2_S_4_/TPA-1-COF. Notably, the photocatalytic activity of H_2_ evolution remains unchanged after 30 h under light irradiation. The integration of MS and COFs in the heterojunction can create synergistic effects, including enhanced transfer separation efficiency, promoted electron transfer, and increased durability of the catalytic active sites. In a core-shell heterostructure, the integration of MS as the core material and COF as the shell material exploits their distinct properties to achieve enhanced functionalities, except for a few studies that have used COFs as the core [81,82]. MS, with its high conductivity and catalytic activity, serves as the robust core that facilitates efficient charge transport and catalytic reactions. Conversely, COF provides a versatile shell with exceptional molecular recognition ability and chemical stability, effectively stabilizing the MS core while modulating its electronic structure and catalytic performance through tailored interactions at the interface. Feng *et al*. [78] prepared covalent CdS@COF core-shell heterojunctions by *in situ* growth of COF protective shells on the surface of CdS nanorods. They systematically investigated the effect of the COF shell thickness on the photocatalytic activity of CdS@COF. The C=N covalent bond formed between the COF shell and –NH_2_ on the surface of CdS nanorods acts as a bridge, providing a channel for rapid electron transfer. This creates a Z-scheme charge transfer path at the interface, preserving strong redox ability. Zhang’s group investigated the functional-group-dependent charge dynamics in Tp-based COF/MgIn_2_S_4_ (MIS) S-scheme heterojunctions for photocatalytic H_2_ evolution [68]. They synthesized two COF variants with the same backbone structure and distinct functional groups, namely, TpPa-1 with H groups and TpPa-2 with –CH_3_ groups. Surface potential analysis confirmed electrostatic-driven heterojunction formation, and contact angle measurements revealed TpPa-1/MIS-5% exhibited superior hydrophilicity with a contact angle of 65.46° compared to 118° for TpPa-2/MIS-5%, thereby enhancing H_2_O adsorption and reaction kinetics. The optimized TpPa-1/MIS-5% achieved a H_2_ production rate of 13.16 mmol g^−1^ h^−1^, which is 4.3 times higher than that of TpPa-2/MIS-5% (3.05 mmol g^−1^ h^−1^). As revealed, the small H groups can promote rapid electron transfer from MIS to TpPa-1 while bulky –CH_3_ groups in TpPa-2 impede this process.

### MX/COF

The unique electron configuration and hybridized band structure of metal ions in MX endow it with excellent photocatalytic performance, garnering much attention in recent years. When integrated with COF, the synergy between the components extends light absorption into the visible and even near-infrared regions, enhances durability against photocorrosion, and enables tailored surface chemistry to optimize reaction kinetics and selectivity [83]. For example, Zhang and co-workers deposited AgBr on the surface of COF TzDa, which was easily reduced to elemental Ag under light irradiation, forming a Z-scheme COF TzDa/Ag/AgBr heterostructure [84]. The composite exhibited a red shift in the UV-vis diffuse reflectance spectra (DRS) with enhanced light absorption compared to AgBr and COF TzDa. Besides, new absorption bands in the 610–720 nm range were observed, attributed to the localized surface plasmonic resonance effect of Ag nanoparticles. Moreover, MX and halide perovskites have been widely applied in photovoltaics, scintillators and other fields due to their efficient light-harvesting capabilities, long excitation lifetimes, high luminous efficiency, considerable photogenerated electron and hole diffusion distances, and precisely tunable band structure. Unlike quantum dots (QDs) that require encapsulation or anhydrous organic solvents for stability, halide perovskite nanocrystals (≥20 nm) with moisture-insensitive exposed surfaces show high competence in solar-driven water-involved chemical reactions [85]. Lin *et al*. [86] developed a nanocrystalline perovskite MAPbBr_3_/COF nanoheterostructure, achieving a 100% coupling yield and an optical quantum efficiency of 11.5%. Such advancement suggests that while halide perovskites are moisture-sensitive, their stability can be improved through exposing moisture-insensitive surfaces. This is crucial for maintaining the performance and longevity of halide perovskite/COF heterojunctions in photocatalytic applications. However, to ensure efficient charge transfer between MX and COF, a major challenge lies in overcoming the significant differences in their crystal structures and chemical properties, in order to achieve a good lattice match at the interface. Moreover, some MX materials are sensitive to humidity and oxygen, requiring strict reaction conditions during their preparation, which increases both complexity and cost.

### g-C_3_N_4_/COF

g-C_3_N_4_ offers several notable advantages, making it highly appealing for photocatalytic applications [87,88]. First, it exhibits a visible-light response. Additionally, g-C_3_N_4_ demonstrates exceptional chemical and thermal stability, preserving its structural integrity and catalytic performance under prolonged light irradiation. Another benefit lies in its straightforward and cost-effective synthesis, relying on inexpensive, readily available organic monomers, which makes it suitable for large-scale production. However, its practical use is hindered by inherent limitations, such as limited light absorption, high carrier recombination rates, and a low number of active sites. To overcome these challenges, researchers are actively exploring the construction of heterostructures to enhance their charge separation efficiency [64,89]. The formation of g-C_3_N_4_/COF heterojunctions is an effective strategy to overcome the key limitations of g-C_3_N_4_. This approach not only significantly mitigates the recombination of photogenerated charge carriers but also enhances the absorption of visible light. The amino groups at the edges of g-C_3_N_4_ readily form imide bonds with aldehydes, resulting in imide-based COF/g-C_3_N_4_ heterojunctions with stronger interactions than those found in inorganic material/COF heterostructures. For example, Xiao *et al*. [90] reported an *in situ* growth method utilizing a Schiff base reaction between g-C_3_N_4_ and aldehyde-functionalized TP-BPY-COF. The formation of imide bonds at the interface between g-C_3_N_4_ and TP-BPY-COF facilitates electron transfer, thus enhancing the overall charge transfer efficiency between the two components. Furthermore, the integration of single-atom platinum via coordination creates a Pt^2+^/Pt^4+^ redox cycle, which aids in both the oxidation and reduction of NO. Additionally, *π*–*π* interactions between COF and g-C_3_N_4_ facilitate efficient interfacial electron transfer. Wang *et al*. [64] developed an S-scheme van der Waals heterostructure composed of g-C_3_N_4_@Tp-Tta COF. Their findings demonstrated that the *π*–*π* interactions are further enhanced by N vacancy in g-C_3_N_4_. This synergy promotes the separation of photogenerated carriers, improving the overall photocatalytic performance.

### MOF/COF

**Figure 4.**
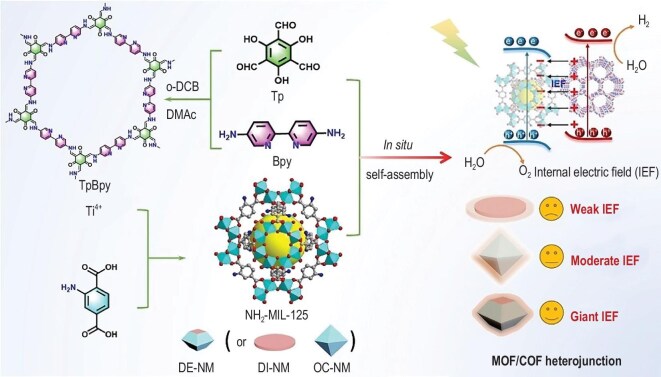
Schematic illustration of the preparation of NH_2_-MIL-125(Ti)/TpBpy-COF [99]. Copyright 2025, Wiley-VCH.

In recent decades, MOFs and COFs have developed rapidly, each achieving diverse structures and compositions through the modulation of parameters such as building units, lattice expansion, connectivity and functionalization strategies [91–94]. Despite their prolific development, the integration of MOFs and COFs into composite materials is appealing. This synergy holds great potential to expand their applications and enhance the understanding of structure–activity relationships. By coupling MOFs and COFs, new structures and functionalities can arise, providing fresh perspectives for material design. For instance, researchers embed exotic metal sites, such as bipyridine- and phthalocyanine-based complexes, into COF frameworks to meet specific functional demands, demonstrating the promise of hybridization to inspire new directions in MOF and COF research [95–97]. To form stable covalent bonds, pretreated MOF (amination) is commonly linked with aldehydes at the terminal groups of COF-conjugated chains through a Schiff base reaction. These MOF/COF hybrids facilitate electron transport and enhance interfacial interactions between the two components. The unique structural confinement and electronic environment of COFs stabilize MOFs against leaching, ensuring sustained photocatalytic activity and selectivity. For example, Hu *et al*. [98] reported a 2D/2D S-scheme heterojunction consisting of NH_2_-Cu-MOF and TPA-1-COF. The size compatibility of the two porous materials resulted in abundant surface reaction sites and strong interactions. Lan’s group developed a covalently linked heterojunction photocatalyst comprising NH_2_-MIL-125(Ti) and TpBpy-COF (Fig. 4) [99]. They rationally controlled the internal electric field in the heterojunction by engineering the crystal facet of the NH_2_-MIL-125(Ti) component, thereby improving charge separation efficiency. This facet-engineered MOF/COF system achieved unprecedented photocatalytic overall water splitting, representing the highest efficiency reported for COF-based photocatalysts. Peng *et al*. [100] used tris(4-formylphenyl)amine (TFPA) to introduce –CHO functional groups onto amine-functionalized rod-like MOF(NH_2_-MIL-68) and grew TPA-COF with a layered, regular pore structure on its surface. This yielded an NH_2_-MIL-68@TPA-COF hybrid material with high crystallinity and a hierarchical pore structure. The hybrid material exhibited permanent porosity, displaying a reversible isotherm with notable hysteresis between the adsorption and desorption curves.

### Polymer/COF

Polymer/COF hybrids combine the flexibility and processability of polymers with the robust structure of COF, offering a versatile platform for photovoltaic and photocatalytic applications. Integrating polymers with COFs enables the tuning of electronic band structures and enhances charge separation efficiency [27]. The ability to modify both polymer and COF components allows for precise control over optical, electronic, and catalytic properties, paving the way for custom functional materials with improved performance and adaptability. Typically, the terminal groups of polymer residues in COF synthesis create specialized interface electron transport channels [101], significantly improving the separation of photogenerated carriers upon heterojunction formation. For example, a 2D/2D Z-scheme heterojunction photocatalyst was successfully constructed through covalent integration of PTO-COF and TpMa-CONs [102]. The synthesis involved a precisely controlled Knoevenagel condensation followed by a ball-milling process, which established covalent linkages between the residual aldehyde groups in PTO-COF and amino groups in TpMa-CONs to facilitate efficient charge transfer. Benefiting from the advantages of a Z-scheme charge transfer mechanism and ultrathin 2D nanostructures, the resulting hybrid material exhibited high activity in visible-light-driven photocatalytic degradation of antibiotics, along with superior stability and reusability performance.

### Others/COF

In addition to the aforementioned strategies, other materials for the construction of COF-based heterojunction photocatalysts include metal phosphate, layered double hydroxide and carbon nanomaterials. For example, BiPO_4_ exhibits limited visible-light absorption, restricting its ability to harness sunlight effectively. Enhancing its light-harvesting capability can be achieved by constructing BiPO_4_@COF composites. Similarly, carbon-based materials like fullerenes (C_60_) can also overcome their inherent limitations of light absorption by heterojunction construction. With a football-like structure composed of 60 carbon atoms arranged in 12 pentagons and 20 hexagons, C_60_ exhibits high electron affinity, excellent chemical stability, and the ability to function as an electron acceptor. He *et al*. [61] synthesized C_60_/TpPa heterojunction materials, leveraging the unique π-orbital surface of C_60_ to facilitate the rapid transfer of photoexcited electrons and suppress charge recombination, thereby improving photocatalytic performance.

## THE SYNTHETIC STRATEGIES FOR COF-BASED HETEROJUNCTION PHOTOCATALYSTS

**Figure 5.**
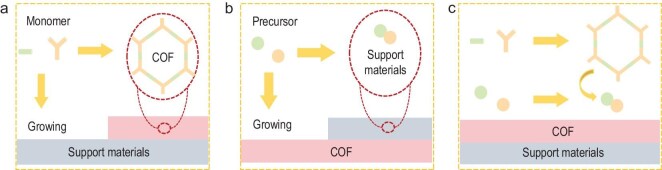
Schematic illustration of synthetic strategies for COF-based heterojunction photocatalysts by (a) COF *in situ* growing on pre-synthesized support materials, (b) support materials *in situ* growing on COF and (c) integrates pre-synthesized individual components.

Synthetic strategies for COF-based composites encompass diverse approaches designed to achieve targeted properties and functionalities. One typical approach is the *in situ* growth of COFs, where COFs are constructed directly from monomers on the surface of a pre-synthesized support (Fig. 5a). Another strategy involves the *in situ* synthesis of the support material, where the support is generated from its precursors directly on a preformed COF, resulting in composites with intimate contact and synergistic properties (Fig. 5b). A third method integrates pre-synthesized components, where the COF and other materials are prepared separately and then combined through techniques such as physical mixing, electrostatic assembly, or other interactions (Fig. 5c). This method promotes the interface interactions between the components by precisely tuning their surface properties. All strategies enhance the stability and efficiency of the heterojunctions by improving interface contact and interfacial charge transfer. The selection of these three strategies depends on factors including the size differences, morphology, and synthetic conditions of the components. This section briefly introduces these different synthesis strategies and the corresponding linkage methods for COF-based composites prepared via aldehyde-amine condensation.

### COF *in situ* growing on pre-synthesized support materials

The combination of covalent connectivity and crystallinity in COF requires special functional groups and carefully controlled reaction conditions, typically designed for reversible covalent bonds. However, with reversible bonding strategies, there is usually a trade-off between achieving high crystallinity and maintaining structural stability. To address this, COFs with fixed morphology and orientation can grow *in situ* by anchoring COF building blocks onto pre-synthesized support materials. This approach ensures strong interactions between COF and the support, thereby enhancing the stability of the composite. For example, *in situ* growth of COF on MO typically involves the solvothermal treatment of COF monomers and MO nanoparticles under synthetic conditions similar to those used for COF formation. TiO_2_@COF-316, featuring a core-shell nanowire array structure, was successfully synthesized by growing and coating COF-316 on the surface of a TiO_2_ core to form the composite [103]. In this system, the Z-scheme charge transfer mechanism allows electrons from TiO_2_ to be injected into the valence band of COF-316, promoting charge separation and accumulating more electrons on the surface of COF-316, thereby enhancing its NO_2_ sensing activity. This structure effectively reverses the properties of TiO_2_ from reducibility to oxidability. Notably, 3-aminopropyltriethoxysilane (APTES) is commonly used to modify the support surface prior to COF growth, strengthening the covalent interaction between COF and MO. Addition or substitution reactions on the surface of the support selectively grafted functional groups, not only altering the intrinsic properties of the composite, such as hydrophobicity and electronegativity, but also increasing the number of active sites, thereby enhancing its reactivity and selectivity. He *et al*. [54] utilized stable NH_2_-MIL-125(Ti) as a substrate for COF growth. The amino functional groups on NH_2_-MIL-125(Ti) provided ample anchoring sites for the subsequent growth of 4,4′,4′′-(1,3,5-triazine-2,4,6-triyl)tribenzaldehyde (TTB-TTA). Initially, NH_2_-MIL-125(Ti) was amino functionalized, and then covalently linked to the TTB-TTA through a direct condensation reaction. Similarly, Peng *et al*. [100] reported a covalently bonded core-shell structure of NH_2_-MIL-68@TPA-COF hybrid material. They first prepared amino-functionalized NH_2_-MIL-68 and then used tris(4-formylphenyl)amine (TFPA) to introduce aldehyde functionality, yielding NH_2_-MIL-68(CHO) with COF seeds on the surface of MOF. Finally, tris(4-aminophenyl)amine (TAPA) was added to condense with TFPA on the surface of MOF, promoting COF growth. Furthermore, the COF-based heterostructure can be constructed through mechanical mixing of the support and monomers, which avoids harsh conditions and saves time.

**Figure 6.**
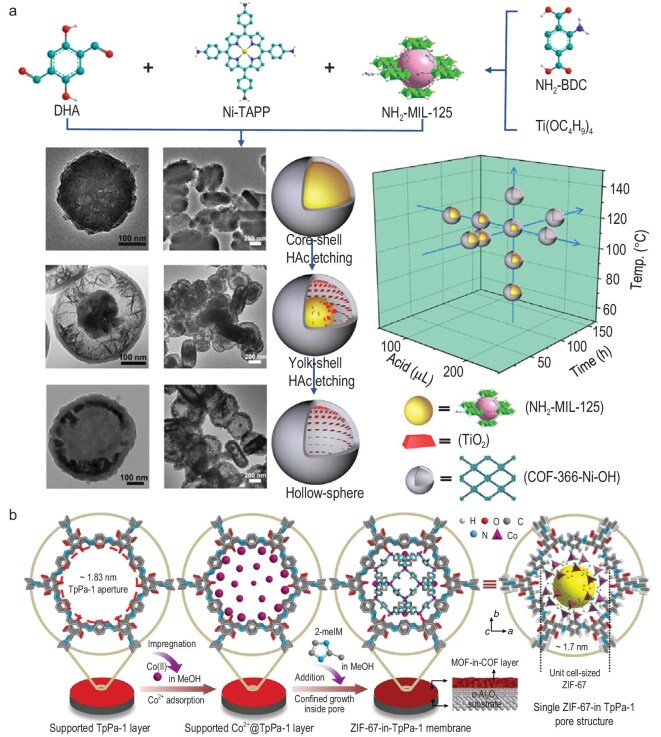
(a) Design principles and fabrication schematics for architecturally diversified COF nanocomposite systems [104]; Copyright 2021, Wiley-VCH. (b) Membrane synthesis flowchart showing ZIF-67 encapsulation in TpPa-1 framework [107]; Copyright 2021, Springer Nature.

In some cases, the support may be too small to meet the growth requirements of COF. To address this limitation, researchers have developed techniques to increase the surface area of the support or employ larger support materials. One approach is to utilize porous materials or nanostructured supports, which provide a higher surface area for COF growth and enhance the interaction between the support and COF. Additionally, employing hierarchical structures or combining multiple supports can create more space and anchor sites for COF formation. These strategies help in achieving uniform and well-dispersed COF-based composites, optimizing their performance for various applications such as catalysis, gas storage, and separation technologies. However, this method typically requires multi-step synthesis processes, which increase operational complexity and time costs. Besides, removing the supports often necessitates the use of extreme conditions such as strong acids, strong bases, or high temperatures, which may destroy the structure of the heterostructure. Recently, Lan’s group reported a MOF-sacrificial *in situ* acid etching strategy for the controlled synthesis of uniform, monodisperse core-shell MOFs@COFs, yolk-shell MOFs/TiO_2_@COFs, and hollow spherical TiO_2_@COFs nanocomposites [104]. As shown in Fig. 6a, unlike other template methods, this strategy eliminates the need for multi-step synthesis or complex template removal processes. By precisely adjusting the acetic acid (HAc) concentration, reaction time, and temperature during synthesis, the MOF core (NH_2_-MIL-125) in the MOFs@COFs undergoes varying degrees of etching, leading to diverse morphologies of COF-based heterostructures while generating nano-TiO_2_. More importantly, NH_2_-MIL-125/TiO_2_@COF-366-Ni-OH-HAc demonstrates high photocatalytic conversion efficiency in a gas–solid mode, owing to its numerous active sites, rapid carrier transfer, and enhanced solar energy utilization.

### *In situ* growing support materials on COF

COF with microporous and 2D structures serve as excellent substrates for the growth of other materials, facilitating the synthesis of COF-based heterostructures [85]. The surface of pre-synthesized COFs typically contains residual hydroxyl groups, which can influence their chemical reactivity, surface hydrophilicity, and interactions with metal ions (M^+^) [105]. However, due to inherent defects, COF faces challenges in forming stable preparative heterojunctions, as most COF linkage bonds are reversible and susceptible to hydrolysis in water and acidic solutions. Prolonged exposure to oxygen can also lead to decomposition, potentially compromising the stability of heterostructures formed by the *in situ* growth of MO on COF. To address these concerns, researchers have considered strategies such as enhancing the chemical stability of COFs by modifying the functional groups at the linkages or by incorporating more robust covalent bond linkages [106]. For COF-based heterojunctions, a viable strategy to enhance stability involves introducing a more rigid substrate and sequentially anchoring COFs and other materials onto its surface to establish stable interface interactions. These approaches are designed to prevent degradation under harsh conditions and enhance electron transfer at the interface, thereby improving the stability, performance, and overall efficiency of COF-based heterojunctions. For metallic compounds, the electrostatic adsorption of M⁺ on COFs is beneficial, allowing COF to effectively anchor M^+^, which can then react with S^2^⁻ and X⁻ to form MS or MX compounds on the COF surface. This approach promotes the formation of interconnected heterostructures. For example, TFPT-DHTH-COF exhibited a negative zeta potential of −16.8 mV at pH 7 in a cadmium acetate aqueous solution [105]. This surface charge facilitated the adsorption of Cd^2^⁺ onto the surface of TFPT-DHTH-COF, followed by the *in situ* growth of CdS nanoparticles on the COF surface with the introduction of S^2^⁻. Similarly, ZnCdS QDs can be grown *in situ* within the 2D TpPa-1-COF to form a 0D/2D ZnCdS/TpPa-1-COF composite, where Cd^2^⁺ and Zn^2^⁺ are first anchored on the surface of the ZnCdS/TpPa-1-COF. Meng and co-workers developed a MOF-in-COF membrane using stable Co-based ZIF-67 and TpPa-1 [107]. They grew a TpPa-1 layer on a porous *α*-Al_2_O_3_ substrate using a solvothermal method (Fig. 6b). The membrane was prepared by immersing the TpPa-1 layer in a Co(NO_3_)_2_·6H_2_O solution to adsorb Co^2^⁺, followed by the introduction of 2-methylimidazole to confine ZIF-67 within the TpPa-1 layer. Scanning electron microscopy (SEM) characterization reveals a continuous ∼1 μm TpPa-1 layer on the *α*-Al_2_O_3_ substrate. After the two-stage immersion process, the membrane retains its original morphology and thickness.

The growth of polymers on COFs relies on the compatibility between COFs and polymer monomers. These materials are typically synthesized under similar reaction conditions, resulting in covalent bond connections during integration. The advantage of polymer/COF composites over other COF-based heterojunctions is that the energy levels of the polymer and COF are linked by covalent bonds rather than mere contact. This connection mode facilitates the recombination of electron-hole pairs with low redox capability, thereby preserving the maximum redox potential of the heterostructure. However, the polymer/COF connection tends to be relatively weak, leading to lower stability during practical photoreactions.

### Integration of pre-synthesized individual components

Apart from the previously mentioned methods, COF-based heterojunctions can be prepared by integrating pre-synthesized individual components through mechanical ball milling, electrostatic self-assembly, and other techniques. The key to synthesizing COF-based heterostructures lies in the precise control of structure and morphology, which ensures that the inherent properties of the individual components remain unchanged. For instance, some MO/COF hybrid materials were assembled from MO and COF dispersed in a solvent in a certain proportion, and a certain temperature is also usually set to improve the stability of this heterojunction. Due to the aromatic nature of MOFs and the long-range ordered structures of COFs, strong *π*–*π* stacking interactions provide a promising method for constructing MOF/COF hybrid materials with tunable properties and enhanced functionalities derived from both parent species. This interaction facilitates precise control over the heterojunction interface, allowing for tailored designs that optimize properties such as electronic band alignment, charge transfer efficiency, and stability under various conditions. For instance, the core-shell structure PCN-222-Co@TpPa-1, featuring Co(II) sites, Zr(IV) cluster Lewis acid sites, and Bronsted base active sites, was prepared through *π*–*π* stacking interactions with TPA-1 [108]. This core-shell structure exhibits high chemical and thermal stability. The *π*–*π* stacking interaction is particularly common in organic polymers or g-C_3_N_4_/COF heterojunctions, where heteroatoms such as N and S can be spatially arranged to enhance interactions between the individual components, promote electron transfer, and reduce transfer distances. Xia *et al*. [109] successfully fabricated a ZnO/covalent triazine framework (CTF) S-scheme heterojunction photocatalyst using a straightforward solid-state mechanical grinding approach. This solvent-free mechanochemical strategy enables tight interfacial contact between the constituent materials while eliminating the need for sophisticated synthesis equipment.

Recently, Zhang *et al*. [70] measured the zeta potentials of TPA-Cl and ZnO at pH 7, determining values of −14.4 mV and 20.8 mV, respectively. By exploiting the opposite surface potentials of these materials, they successfully fabricated a TpPa-Cl/ZnO S-scheme heterojunction using electrostatic self-assembly. This method leverages the complementary surface charges to create a structured interface, enhancing the potential for applications in advanced materials and catalysis. Additionally, a unique COF/QDs S-scheme heterojunction composite photocatalyst was constructed by anchoring CsPbBr_3_ QDs on TPA-COF nanoplates through an electrostatic self-assembly strategy [39]. TPA-COF exhibited a distinct lamellar morphology and high crystallinity, while the CsPbBr_3_ QDs, characterized by a cubic phase and particle sizes of ∼4–10 nm, were primarily deposited within the COF pore.

## APPLICATIONS OF COF-BASED HETEROJUNCTION PHOTOCATALYSTS

COF-based heterojunction photocatalysts offer significant promise for a wide range of applications, leveraging their substantial specific surface area, excellent light absorption and adjustable band structure. These materials combine the unique properties of conjugated organic COFs with other semiconductor materials to enhance performance in solar-driven catalytic processes. COFs, constructed from conjugated organic molecules linked by covalent bonds, allow for precise control over pore structures and chemical functionalities. Their strong visible-light absorption capabilities make them highly suited for boosting solar energy conversion efficiency. Integrating COF with materials like MO and MS, these heterojunctions effectively promote charge separation and improve carrier utilization, thus enhancing catalytic activity. This section systematically reviews the applications of COF-based heterojunction photocatalysts in pollutant photodegradation, photocatalytic H_2_ evolution, CO_2_ reduction, H_2_O_2_ production and organic synthesis [110]. Emphasis is placed on exploring the synergistic interactions between COFs and other materials and the underlying mechanisms of photoredox reactions.

### Pollutant photodegradation

With accelerated global industrialization and urbanization, substantial amounts of organic pollutants, heavy metals, and other hazardous substances are discharged into soils, water bodies, and the atmosphere, posing threats to ecosystem stability and human health [111–113]. Traditional pollutant treatment methods such as chemical oxidation, adsorption, and biological treatments, encounter challenges, including high energy consumption, low treatment efficiency, and difficulties in managing by-products. Photocatalytic pollutant degradation harnesses solar energy to activate catalyst surfaces and generate excitons that initiate redox reactions, decomposing pollutants into harmless or less toxic substances. COF-based heterojunction photocatalysts with substantial specific surface area, excellent light absorption, tunable structures and redox capabilities could greatly enhance photocatalytic degradation processes.

Bi *et al*. [114] evaluated the photocatalytic performance of Ti-MOF@DATp using the tetracycline (TC)-U(VI) mixture system as a target pollutant, which was a necessary condition for water purification. Compared to the removal of U(VI) alone, the equilibrium time reduced from 90 to 30 min in a TC-U mixture system, while the removal efficiency increased from 89% to 96%. Similarly, TC removal efficiency in the mixture system increased from 77% to 90% compared to TC removal. This enhancement can be attributed to the DATp component in Ti-MOF@DATp, which allows TC to specifically capture U(VI) through interactions with Ti-MOF. Besides, the Z-scheme heterojunction constructed by a covalent bond between Ti-MOF and DATp enhanced the light absorption ability while maintaining high redox capability, thus improving photocatalytic efficiency. Zhang and co-workers investigated the effect of MoS_2_/COF on the degradation of TC and rhodamine B (RhB) and discovered that the degradation efficiency could reach up to 98% and 85.9%, respectively, which was about 3- and 4-fold higher than those of individual components [115]. Trapping experiments showed that the photogenerated hole contributed primarily to the decomposition of RhB.

To evaluate the effect of COF structure on the photocatalytic performance over g-C_3_N_4_/COF composites, Guo *et al*. [50] prepared three composites by substituting the H atom on *p*-phenyldiformaldehyde (TP) with an electron-donating –OCH_3_ and electron-deficient F atom. The introduction of –OCH_3_ resulted in a more widespread distribution of electrons in CB of 1,3,5-tris(4-aminophenyl)benzene (TPB)-TP-OCH_3_, primarily within the TPB moiety. This distribution facilitated the transfer of electrons from g-C_3_N_4_ to TPB-TP-COFs in the heterostructure. In contrast, the electrons in CB of TPB-TP-F were mainly localized in the TP moiety after F atom substitution, which hinders electron transfer. Consequently, the phenol degradation performance of TPB-TP-OCH_3_ outperformed that of TPB-TP-F.

A new configuration gaining attraction in recently proposed organic-inorganic N-COF/BiOBr S-scheme heterojunctions involves assembling hollow spheres into a sphere-nanosheet-like flake heterostructure [116]. This heterostructure was fabricated through the *in situ* synthesis of BiOBr from molecular building blocks on the surface of the pre-synthesized N-COF hollow sphere. The resulting heterostructures displayed notable characteristics, including close contact and numerous reactive sites on their surface. After 120 min light irradiation, the TC concentration decreased by only 28% and 15% for N-COF and BiOBr, respectively. The optimal 20N-COF/BiOBr improved the degradation efficiency of TC, achieving ∼81.2%. This enhanced efficiency arises from the S-scheme charge transfer mechanism between N-COF and BiOBr, maximizing the redox capacity.

A nanocomposite ZnAgInS/COF with type-II heterostructure was prepared using a straightforward oil bath heating method [117]. SEM and transmission electron microscopy (TEM) images of ZnAgInS/COF revealed that the ZnAgInS microspheres were evenly distributed across the surface of TpPa-1 COF nanorods, creating close interfacial contact to enable efficient charge transfer between the interfaces. Under simulated solar light irradiation, the optimal ZnAgInS/COF composite achieved an impressive RhB (60 mg/L) degradation efficiency of ∼98% within 60 min, whereas the degradation efficiencies of TpPa-1 COF, ZnAgInS, and a physically mixed sample were only 12%, 54% and 67%, respectively. The apparent rate constant of ZnAgInS/COF(5) was measured to be 0.06659 min^−1^, which is 5 times, 30 times and 3 times higher than that of ZnAgInS, TpPa-1 COF, and the physically mixed sample, respectively. In a related study, Zhang *et al*. [118] synthesized a direct Z-scheme MOF@COF heterojunction through covalent linkage. Zr-MOF@TzDa-COF composite exhibited the strongest photocurrent response and the smallest arc radius, indicating rapid carrier transfer between the covalently connected Zr-MOF and TzDa-COF. Trapping experiments identified ^•^O_2_^−^ and ^•^OH as the primary active species in the degradation of potassium butyl xanthate (PBX). However, the oxidation capacity of TzDa-COF was insufficient to produce ^•^OH, which contradicted the apparent ^•^OH signal in the heterostructure. Therefore, the photogenerated electron and hole with the strongest redox capacity were retained, which accorded with the Z-scheme charge transfer mechanism.

MOF selectively binds different metal ions at their unsaturated metal sites, while Mg-TC complexes exhibit specific fluorescence characteristics, giving Mg-MOF unique optical and fluorescence properties. Yang *et al*. [119] deposited high crystallinity TPA-1-COF and Mg@Fe-MIL-101 successively on stainless steel mesh using a two-step solvothermal method. Mg^2+^ modified Fe-MIL-101 through metal substitution, enhanced the light absorption range of Mg@Fe-MIL-101/TPA-1-COF and facilitated effective charge separation. This modification improved the TC degradation rate of Mg@Fe-MIL-101/TPA-1-COF composite (0.0497 min⁻^1^). Meanwhile, unsaturated metal sites in Mg@Fe-MIL-101 can serve as an indicator of TC fluorescence detection by forming Mg-TC complexes. The photocatalytic degradation over COF-based heterojunction photocatalyst is summarized in Table 1.

**Table 1.** Summary of recent representative studies on COF-based heterojunction photocatalysts for pollutant photodegradation.

	Light source				
Compound	Filter (nm)	Power (W)	Pollutant/Concentration	Efficiency	Ref.
MOF@DATp	>300	300 (Xe)	U (300 mg L⁻^1^) TC (30 mg L⁻^1^)	96% (30 min) 90% (30 min)	[114]
MoS_2_/COF	AM 1.5	300 (Xe)	TC (30 mg L⁻^1^) RhB (30 mg L⁻^1^)	98% (30 min) 85.9% (30 min)	[115]
TPB-TP-OCH_3_	>300	100 (LED)	Phenol (10 mg L⁻^1^)	84.8% (120 min)	[50]
20N-COF/BOB	>420	300 (Xe)	TC (60 mg L⁻^1^)	81.2% (120 min)	[116]
ZnAgInS/COF(5)	AM 1.5	300 (Xe)	TC (60 mg L⁻^1^) RhB (60 mg L⁻^1^)	90% (60 min) 98% (60 min)	[117]
Zr-MOF@TzDa-COF	>420	300 (Xe)	PBX (20 mg L⁻^1^)	95% (40 min)	[118]
Mg@Fe-MIL-101/TPA-1-COF	>420	300 (Xe)	TC (100 mg L⁻^1^)	98% (80 min)	[119]
Ag_3_PO_4_/COF	>420	300 (Xe)	Pymetrozine (100 mg L⁻^1^) RhB (15 mg L⁻^1^)	99.3% (40 min) 96.7% (40 min)	[120]
COF TzDa/Ag/AgBr	>400	300 (Xe)	TC (10 mg L⁻^1^)	80.2% (30 min)	[84]
BTDC	>400	300 (Xe)	RhB (10 mg L⁻^1^)	98% (30 min)	[48]
CuPor-Ph-COF/g-C_3_N_4_	>400	300 (Xe)	RhB (20 mg L⁻^1^)	94.5% (90 min)	[87]
In_2_S_3_/COF(3)	AM 1.5	300 (Xe)	TC (100 mg L⁻^1^) RhB (100 mg L⁻^1^)	92% (60 min) 77.6% (120 min)	[121]
HT-3	>400	300 (Xe)	Acetaminophen (10 mg L⁻^1^)	99% (60 min)	[122]
10TDCN	>300	300 (Xe)	RhB (10 mg L⁻^1^)	100% (45 min)	[123]

### H_2_ evolution

Solar-driven water splitting is an ideal approach for achieving clean energy production [124–130]. Since Fujishima and Honda first reported photocatalytic H_2_ production using TiO_2_ electrodes in 1972 [131], researchers have devoted numerous photocatalysts to improving solar-to-hydrogen conversion efficiency [132–139]. Highly efficient hydrogen production photocatalysts typically possess a broad range of light absorption, effective charge separation, abundant active sites, and strong reducing abilities. COF-based heterojunctions have attracted much attention due to their excellent light-harvesting capacity, efficient charge separation and large specific surface area [76,89,140]. Besides, the inherent porosity of COFs and their large conjugated skeleton provide a location for the accommodation of catalytic sites.

In 2014, Kurungot and co-workers utilized COF(TpPa-2) as a substrate to anchor CdS nanoparticles, marking the first instance of COF-based heterostructures applied to photocatalytic H_2_ production [141]. The results indicated that with 10% COF content, the H_2_ production rate (HER) of the hybrid reached 3678 μmol g⁻^1^ h⁻^1^ under lactic acid (LA) solution, which was ∼29 times higher than that of bulk CdS. This composite featured a conjugated skeleton structure, a large specific surface area, and abundant ordered 2D heterointerfaces, all of which contributed to stable photogenerated electrons and resulted in excellent photocatalytic activity. However, the type-II charge transfer mechanism between CdS and COF(TpPa-2) failed to reduce the photocorrosion of CdS in the heterostructure. A more effective configuration, the recently reported core-shell heterojunction (TCOF@CdS), was prepared through *in situ* self-polymerization of 1,3,5-benzenetricarboxaldehyde and 2,4,6-tri(4-aminophenyl)-1,3,5-triazine on the CdS nanosphere [142]. *In situ* X-ray photoelectron spectroscopy (XPS) confirmed that the charge transport paths of COF and CdS conform to a Z-scheme mechanism. Besides, the *W*_f_ of CdS and T-COF measured by ultraviolet photoelectron spectroscopy were 4.68 and 4.89 eV, respectively. This indicated that the electrons from T-COF recombined with the holes of CdS at the interface under light irradiation. Consequently, T-COF serves as a protective shell that safeguards the H_2_ production center (CdS) from deactivation while also acting as an oxidation site to prevent photocorrosion of CdS due to photogenerated holes. Additionally, the C–S bond formed between T-COF and CdS provided a stable channel for rapid charge transfer. T-COF@CdS-3 exhibited the highest HER of 12.5 mmol g⁻^1^ h⁻^1^ under full spectrum irradiation, with an apparent quantum yield (AQY) of 37.8% at 365 nm, surpassing most CdS-based composite materials. Therefore, constructing Z-scheme or S-scheme MS/COF heterostructures is expected to be an effective strategy for inhibiting photocorrosion of MS, where MS is often employed as a reduced semiconductor in MS/COF heterostructures.

Introducing donor-acceptor (D-A) into COF is an effective strategy to improve photocatalytic performance. In this configuration of COF-based heterostructures, the synergistic effect of the molecular polarization of the D-A moiety and the interfacial interaction between different semiconductors improves electron mobility. Shen *et al*. [134] synthesized a D-A COF using 2,4,6-trihydroxybenzene-1,3,5-tricarbaldehyde and 3,7-diaminodibenzo[b,d] thiophene 5,5-dioxide (TSO) through a photo-assisted method, and grew it *in situ* on WO_3_ nanosheets (labelled as TSCOFW) to form a 2D/2D heterojunction. In TSCOFW, the Z-scheme charge transfer mechanism facilitates the accumulation of more electrons on COF. Due to the electron-deficient properties of TSO, photogenerated electrons predominantly accumulate on the TSO moiety. The O atoms on TSO can form hydrogen bonds with H_2_O molecules, acting as an electron output ‘tentacle’ that promotes further electron transfer to H_2_O. The TSCOFW exhibited a nearly molar-scale HER of 593 mmol g⁻^1^ h⁻^1^. This ultra-high H_2_ production activity arises from the synergistic effect of the built-in electric field at the Z-scheme heterojunction interface, the strong water oxidation ability of WO_3_, and the effective electron output of TSO. Notably, TSCOFW achieved an HER of 146 μmol g⁻^1^ h⁻^1^ and an O_2_ evolution rate of 68 μmol g⁻^1^ h⁻^1^ without any sacrificial agent, surpassing other COF-based heterojunction photocatalysts in pure H_2_O. In addition to TSO moiety, pyrene (Py) has recently been utilized in the design of COF-based heterojunctions. Py possesses a highly π-conjugated system and a rigid structure, which helps minimize non-ideal torsion and vibration in the COF-conjugated chains, thereby maintaining electron orbital alignment and improving the electron transfer within COF. A D-A COF/g-C_3_N_4_ heterojunction was prepared by combining COF (benzothiadiazole-*alt*-Py) with 2D g-C_3_N_4_ through *π*–*π* interactions [89]. Compared to pure g-C_3_N_4_, the uniquely designed D-A COF composite exhibited good visible-light absorption and sensitizer functionality, with *π*–*π* interactions between the two components facilitating the separation and migration of photogenerated carriers. COF/g-C_3_N_4_ showed an HER of 27.54 mmol g⁻^1^ h⁻^1^ under visible light irradiation, with an AQY of 15.5% at 420 nm. Wang *et al*. synthesized another D-A COF by employing thiophene groups as electron donors and imine groups as electron acceptors via a Schiff base reaction, and integrated it *in situ* with snowflake-shaped CdS exposing the (002) crystal plane in order to construct an organic-inorganic semiconductor heterojunction [143]. This design leverages intramolecular and interfacial built-in electric fields to enhance photo-generated carrier mobility, achieving a 5-fold enhancement in HER performance compared to pristine CdS under visible light irradiation.

COF is rich in N atoms, which can effectively anchor M^+^ and promote the formation of M–N bonds within MOF/COF heterojunctions [54,144], enhancing electron transport efficiency and boosting photocatalytic H_2_ production. A series of multivariate (MTV)-Ti-MOF/COF hybrid materials, PdTCPP⊂PCN-415(NH_2_)/TpPa, were developed through a covalent integration strategy [145]. These composites exhibited good visible light utilization, suitable bandgap and high surface areas conducive to photocatalytic H_2_ production. MTV-Ti-MOF combines with an iminyl-bond COF to form ultra-stable keto-amine moieties. The optimal composite achieved a maximum HER of 13.98 mmol g⁻^1^ h⁻^1^ and a turnover frequency of 227 h⁻^1^, outperforming pure PdTCPP⊂PCN-415(NH_2_) (0.21 mmol g⁻^1^ h⁻^1^) and TpPa (6.51 mmol g⁻^1^ h⁻^1^). Lan *et al*. [146] developed a contently linked NH_2_-UiO-66/TpPa-1-COF heterostructure via a one-pot method. This covalent linkage enhanced the stability of the composite material and promoted the efficient transfer of photogenerated electrons across the interface. The composite displayed a similar light absorption range to TpPa-1-COF, which enhanced the light utilization of NH_2_-UiO-66. Besides, NH_2_-UiO-66/TpPa-1-COF exhibited a relatively small semicircle radius compared to TpPa-1-COF, indicating lower electron-transfer resistance within the heterostructure. The transient photocurrent of the composite was ∼3.5 times higher than that of pure TpPa-1-COF, demonstrating enhanced charge carrier migration. These results confirmed that the covalent link between NH_2_-UiO-66 and TPA-1-COF effectively promotes charge carrier separation. The hybrid material achieved a peak HER of 23.41 mmol g⁻^1^ h⁻^1^, which was 20 times higher than that of TpPa-1-COF, and it maintained stable H_2_ evolution performance over 480 h.

**Figure 7.**
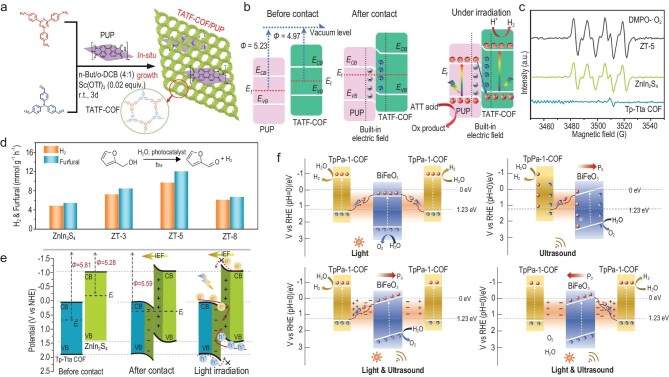
(a) The synthetic process for the TATF-COF/PUP heterostructures. (b) The formation of TATF-COF/PUP S-scheme heterojunction and the proposed charge transfer mechanism [65]; Copyright 2022, Elsevier. (c) DMPO-^•^O_2_^−^ signal over ZnIn_2_S_4_, Tp-Tta COF and ZT-5 under light irradiation. (d) H_2_ evolution and furfural production rates over as-prepared samples. (e) The formation of TATF-ZnIn_2_S_4_/Tp-Tta COF S-scheme heterojunction and the proposed charge transfer mechanism [149]; Copyright 2012, The Royal Society of Chemistry. (f) Schematic diagram of photocatalysis, piezocatalysis and piezo-photocatalysis with different directions of polarization for BiFeO_3_@TpPa-1-COF [147]; Copyright 2022, Wiley-VCH.

Compared to other COF-based heterojunctions, research on polymer/COF hybrids for photocatalytic H_2_ evolution is relatively scarce. This might stem from the high exciton binding energy and hydrophobic nature of such materials, which are less favorable for H_2_ evolution from H_2_O. Li and co-workers tried to address these challenges by constructing an S-scheme TATF-COF/perylene diimide urea polymer (PUP) composite through *in situ* coupling of a 2D triazine‐based imine-linked TATF-COF with PUP, effectively reducing the exciton binding energy in the all-organic system (Fig. 7a) [65]. As shown in Fig. 7b, due to the different *W*_f_ of TATF-COF and PUP, electrons flowed from TATF-COF to PUP after contact until the *E*_f_ reached equilibrium. This contact-induced band bending in semiconductors forms a built-in electric field directed toward PUP at the interface. This electric field drove the migration and recombination of the weakly reducing electron in the CB of PUP with the oxidative hole in the VB of TATF-COF across the interface, while the electron and hole with stronger redox capability were retained. This S-scheme heterojunction enabled efficient carrier separation, maximized the redox capability, and enhanced the performance, achieving 94.5 mmol g⁻^1^ h⁻^1^.

Although a large number of COF-based heterostructures have been developed for photocatalytic H_2_ production and demonstrated impressive activity, many of these systems require sacrificial agents such as LA, ascorbic acid (AA) and triethanolamine (TEOA) and operate under vacuum or inert gas [147,148]. The value of produced H_2_ usually cannot offset the cost of the added sacrificial agent. Therefore, integrating photocatalytic H_2_ evolution with the organic oxidation reactions offers a way to improve overall photoelectron utilization efficiency while generating value-added products. Liu and co-workers developed a ZnIn_2_S_4_/Tp-Tta hybrid material featuring a hierarchical sandwich-like structure via *in situ* growth of Znln_2_S_4_ nanosheets on a Tp-Tta COF nanoplate [149]. This unique structure enhanced light utilization efficiency. Electron paramagnetic resonance results showed a stronger DMPO-^•^O_2_^−^ signal in the composite than in ZnIn_2_S_4_ and Tp-Tta COF, indicating the strong reduction ability in the ZnIn_2_S_4_/Tp-Tta composite, and thus proving the S-scheme charge transfer mechanism (Fig. 7c). The photocatalytic performance of the composite for H_2_ production was systematically evaluated in the furfural solution. As depicted in Fig. 7d, the optimal hybrid material achieved an HER of 9.73 mmol g⁻^1^ h⁻^1^ with a corresponding furfuryl alcohol oxidation rate of 12.1 mmol g⁻^1^ h⁻^1^. The difference between the oxidation potential of furfuryl alcohol and the VB of COF served as the driving force for oxidation. This enhanced activity underscores the strong redox capacity of the S-scheme heterostructure as shown in Fig. 7e.

Recently, an integrated mode has applied the unique electric field regulation capabilities of piezoelectric catalysis, combined with light absorption and electron transport properties of photocatalytic materials, to improve HER [147]. A BiFeO_3_@TpPa-1-COF core-shell hybrid material was constructed by *in situ* growth of TPA-1-COF on BiFeO_3_ nanoparticles. This is the first time that covalent bonds have been reported to integrate COF with piezoelectric materials. This synergy between the internal electric field generated by polarization potential and the optically induced charge carriers improves the activation of active sites for complete water splitting. Under the combined action of light and ultrasound, illumination provides sufficient charge carriers through ultrasonic vibration to establish an alternating built-in piezoelectric field (Fig. 7f). This piezoelectric field, with its adjustable polarization direction, precisely controls hole movement, enhancing charge separation within the BiFeO_3_@TpPa-1-COF composite. In this setup, TpPa-1-COF effectively traps electrons, while BiFeO_3_ preferentially collects holes, further accelerating charge separation and contributing to improved photocatalytic efficiency. The optimized hybrid material reached remarkable H_2_ and O_2_ evolution rates of 1416.4 and 708.2 μmol g^−1^ h^−1^, respectively, under combined ultrasonication and light irradiation. Theoretical calculations indicated that the piezoelectric effect reduces the reaction barrier in the BiFeO_3_@TpPa-1-COF heterostructure, enhancing its overall water-splitting activity. The photocatalytic H_2_ production of a COF-based heterojunction photocatalyst is summarized in Table 2.

**Table 2.** Summary of recent representative studies on COF-based heterojunction photocatalysts for photocatalytic H_2_ evolution.

	Light source					
Compound	Filter (nm)	Power (W)	Sacrificial agent	Activity (mmol g^−1^ h^−1^)	AQY (%)	Ref.
COF(TpPa-2)	>420	300 (Xe)	LA	3.68	4.2	[141]
T-COF@CdS-3	>420	300 (Xe)	AA	12.5	38.7	[142]
TSCOFW	>420	300 (Xe)	TEOA	593	56.1	[134]
NMS/SCN	AM 1.5	300 (Xe)	AA	27.5	15.5	[89]
PdTCPP⊂PCN-415(NH_2_)/TpPa	>400	300 (Xe)	AA	14.0	5.9	[145]
NH_2_-UiO-66/TpPa-1-COF	>420	300 (Xe)	AA	23.4		[146]
TATF‐COF/PUP	>420	350 (Xe)	AA	94.5	19.7	[65]
ZT-5	=400	80 (LED)	Furfuryl alcohol	9.7	13.9	[149]
BFO@COF20-C	>420	300 (Xe)		1.4	0.8	[147]
rGO/BP/TpPa-1	>420	300 (Xe)	AA	18.1	1.3	[150]
COF/CN	>400	300 (Xe)	TEOA	13.4		[140]
COF–CN (1:10)	>420	300 (Xe)	AA	12.8	15.1	[88]
CdS-1%COF	>420	300 (Xe)	AA	15.1	23.9	[151]
CdS@TTI-COF	>400	300 (Xe)	Na_2_S/Na_2_SO_3_	2.8		[78]
COF/CTF	>420	300 (Xe)	TEOA	14.1		[27]
α-Fe_2_O_3_/TpPa-2-COF	>420	300 (Xe)	AA	3.8		[63]
TPCNNS‐2	>420	300 (Xe)	AA	1.1		[152]
BDA-THTA-30	>420	300 (Xe)	AA	9.7	1.6	[153]
In_2_S_3_@HLZU-1	>420	300 (Xe)	Na_2_S	11.9		[154]
NCM/TP1C	>320	300 (Xe)	AA	4.2	0.72	[98]
Cu-NH_2_-MIL-125/TpPa-2-COF	>420	300 (Xe)	Sodium ascorbate	9.2	8.6	[155]
TpPa-2-COF/SnNb_2_O_6_	>420	300 (Xe)	Methanol	0.8		[156]
SnS_2_/TpPa-1-COF	>420	300 (Xe)	AA	2.7	0.23	[45]
COF-TpPa-1/TiO_2_	>420	300 (Xe)	AA	1.4		[157]
TiO_2-x_/TpPa-1-COF	>420	300 (Xe)	Sodium ascorbate	15.3	6.7	[158]
ZnCdS/TpPa-1-COF	>420	300 (Xe)	Na_2_S/Na_2_SO_3_	6.2	0.3	[46]
TpPa-1/MIS-5%	>420	300 (Xe)	Sodium ascorbate	13.16	5	[68]
NH_2_-MIL-125(Ti)/TpBpy-COF	>420	300 (Xe)		0.33	6.06	[99]
COF-Qui-TiO_2_	>420	300 (Xe)	Methanol	5.37		[159]
CT-11	>420	300 (Xe)	AA	37.4	5.91	[160]
CC-15	>420	10 (LED)	TEOA	11.2	1.21	[161]
CC20	>420	5 (LED)	LA	5.2	0.45	[143]

### CO_2_ reduction

Solar-driven conversion of CO_2_ into valuable chemicals or fuels presents a promising solution to global energy demands and environmental challenges [162–167]. COFs have garnered considerable attention due to their highly controllable structures, porous properties, and diverse functional groups. In particular, residual –NH_2_ groups and inherent pore structures within COFs substantially enhance CO_2_ adsorption, positioning them as promising candidates for efficient photocatalysts [168]. However, single COF photocatalysts face challenges in photocatalytic CO_2_ conversion, including insufficient catalytic activity and stability for practical applications. To overcome these limitations, researchers are increasingly exploring the integration of COFs into heterostructures, to enhance charge separation efficiency through interfacial effects.

In 2020, Zhang *et al*. pioneered the study of photocatalytic CO_2_ reduction using a MO/COF heterostructure [169]. These catalysts integrate water-oxidizing active semiconductors (TiO_2_, Bi_2_WO_6_, *α*-Fe_2_O_3_) with COFs (COF-316/318). The Mot-Schottky curve test results indicated that the CB positions of COF-318, TiO_2_, Bi_2_WO_6_ and *α*-Fe_2_O_3_ were −0.75, −0.55, −0.48 and −0.46 eV, respectively. Based on their band structure, the positions of VB were determined to be 0.86, 2.62, 2.34 and 1.40 eV, respectively. The Z-scheme charge transfer mechanism was further verified by the reverse electron migration observed in XPS under UV-light irradiation, with COF-318 and TiO_2_ exhibiting a staggered band structure. The optimal CO generation rate of COF-318-TiO_2_ is 278.7 µmol g^−1^, which is much higher than that of COF-318 (53.2 µmol g^−1^) and TiO_2_ (47.2 µmol g^−1^). Importantly, the performance of the physically mixed COF-318/TiO_2_ was only 117.2 µmol g^−1^, highlighting the advantage of covalent bonding between heterojunctions. To prevent the MO nanoparticles from destroying the porous structure and electron delocalization channel of the COF substrate during the growth process, Kim and co-workers proposed a pore wall modification strategy to achieve an orderly arrangement of ultrasmall TiO_2_ nanodots (≈1.82 nm) within the pores of COF through site-specific nucleation [170]. This strategy stabilized TiO_2_ nanoparticles and facilitated their *in situ* growth on the COF. Specifically, site-specific nucleation occurred between the organic wall of the metal precursor and COF, where the strongly bonded metal precursor restricted the growth of TiO_2_ nanodots during subsequent hydrolysis. Compared to simple pore-filled benzidine (BD)-COF-TiO_2_, TiO_2_ nanodots on the site-specific nucleated heterostructure BD-COF-TiO_2_ showed a smaller particle size, indicating that TiO_2_ nanodots were confined within the COF matrix. The stronger interaction between COF and TiO_2_ (N-Ti-O) resulted in effective electron transfer and enhanced photocatalytic CO_2_ reduction performance. 2,2′-Bipyridine-5,5′-diamine (BPDA)-COF-TiO_2_ composite achieved a CO production rate of 91 µmol g⁻^1^ h⁻^1^, approximately twice of that obtained with the simple pore-filled heterostructure BD-COF-TiO_2_. Similarly, Cheng *et al*. developed a 3D/0D S-scheme heterojunction photocatalyst by spatially confining ZnSe QDs within the porous cages of the 3D COF, enabling coupled photocatalytic CO_2_ reduction and value-added chemical synthesis [171]. The COF porous cages act as nanoreactors, promoting the mass transfer of reactants, while the S-scheme heterojunction promotes directional charge transfer and efficient charge separation. The optimal COF/ZnSe hybrid exhibits a CO production rate of 128.3 μmol g⁻^1^ h⁻^1^, while simultaneously achieving a 95.1% conversion of 1-phenylethanol to 1-phenylethanone.

In solid–liquid systems, the performance of CO_2_ reduction can be improved by adding sacrificial agents and photosensitizers. Common sacrificial agents include TEOA, AA and 1,3-dimethyl-2-phenyl-2,3-dihydro-1H-benzimidazole, while a typical photosensitizer is Ru(bpy)_3_^2+^ [172]. It is worth noting that both sacrificial agents and photosensitizers can independently reduce CO_2_ to CO under certain conditions. Therefore, to prevent misinterpretation of photocatalytic performance, isotope experiments and blank control tests must be conducted under the same conditions, using only the sacrificial agent or photosensitizer, to rule out false-positive results. Yang *et al*. [75] developed MoS_2_@COF composites by modifying MoS_2_ with amino groups and incorporating it into the COF. Both experimental and theoretical results demonstrated strong electron interactions between individual components, enhancing the separation and transfer of interfacial charges and resulting in superior photocatalytic CO_2_ reduction activity under visible light. Upon illumination, the MoS_2_@COF composite achieved a C_2_H_6_ production rate of 56.2 μmol g⁻^1^ h⁻^1^ in the presence of TEOA and Ru(bpy)_3_^2+^, which was 8.6 times and 31.2 times higher than those of pure MoS_2_ and COF, respectively.

Porphyrins can absorb visible and near-infrared light due to their unique electron-rich properties, which can also facilitate the charge separation of COF-based heterostructures. Quach *et al*. [173] synthesized a series of UiO-66-NH_2_/COF-366-Co composites with varying UiO-66-NH_2_ content. A new peak at 450 nm appeared in the composite, indicating a synergistic interaction between COF and MOF, which led to an overall enhancement of its optical properties. Additionally, the reduced peak observed at 710 nm corresponds to the crystal field transition within the system, suggesting effective interactions between Co in COF and UiO-66-NH_2_. The optimized COF-366-Co/UiO-66-NH_2_ composite demonstrated improved efficiency in CO_2_ photoreduction, achieving ∼4092.2 µmol g⁻^1^ h⁻^1^ of CO production with 73.3% selectivity after 4 h, which was 2.4-fold higher than that of bare COF. Similarly, another work of porphyrin-based MOFs@COF composite has been reported by Huang and co-workers [174]. The porphyrin-based Pro-COF-Br shells were grown *in situ* on a NH_2_-UO-66 core, with abundant free amino groups on the surface of the core covalently linked to the shell via a Schiff base reaction. The results indicated that the integration of the porphyrin structure into the shell allowed the heterostructure to absorb light across both UV and visible regions. This enhanced light absorption of the hybrid material resulted in an impressive CO yield of 104.86 mmol g⁻^1^, surpassing Zr-MOF (42.42 mmol g⁻^1^) by ∼2.5 times and Pro-COF-Br (30.21 mmol g⁻^1^) by 3.5 times.

**Figure 8.**
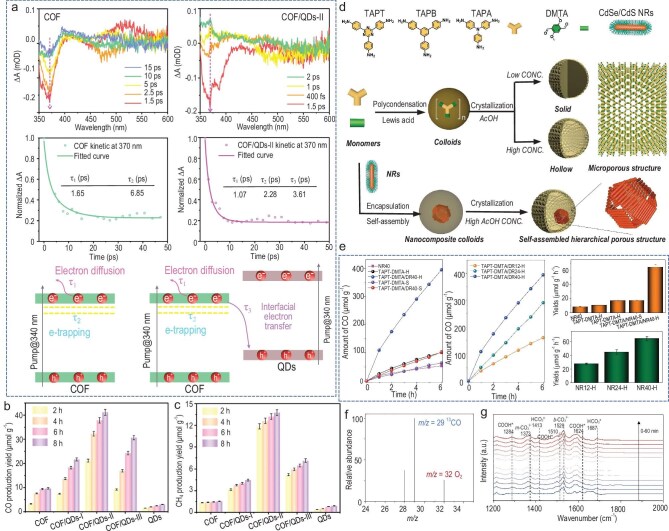
(a) Fs-TA spectra recorded at indicated delay time, normalized decay kinetic curves and charge transfer pathways of samples. Photocatalytic CO_2_ reduction to (b) CO and (c) CH_4_ products over as-prepared catalysts during the 8 h experiment under UV-visible light irradiation [39]; Copyright 2024, American Chemical Society. (d) The synthetic process for TAPT-DMTA/NRs heterostructures. (e) Photocatalytic CO_2_ reduction activity of as-prepared samples. (f) GC-MS analysis of ^13^CO_2_ isotope after ^13^CO_2_ photoreduction in the presence of the composite. (g) The *in situ* DRIFT spectra of TAPT-DMTA/NR40-H in the presence of CO_2_ and H_2_O vapor within 60 min [175]; Copyright 2022, Springer Nature.

Recently, He *et al*. [39] demonstrated that anchoring CsPbBr_3_ QDs onto COF can enhance CO_2_ adsorption and activation within the heterostructure. They investigated S-scheme charge transfer dynamics in COF/QDs nanostructures by femtosecond transient absorption spectroscopy (fs-TA). Both pure COF and COF/QDs composites exhibited a negative signal at ∼370 nm, attributed to the ground state bleaching of photoelectrons in COF (Fig. 8a). The normalized decay kinetics at 370 nm revealed that the lifetimes *τ*_1_ and *τ*_2_ of pure COF were 1.65 and 6.85 ps, respectively, whereas these lifetimes were shortened to 1.07 and 2.28 ps in COF/QDs composites. Moreover, an extra lifetime *τ*_3_ was detected in COF/QDs, which was ascribed to interfacial electron transfer. These results showed that the integration of CsPbBr_3_ and COF provided a new electron transfer channel, effectively shortening the diffusion and capture lifetime of photogenerated electrons and verifying the S-scheme charge transfer mechanism in the COF/CsPbBr_3_ heterostructure. The optimal COF/QDs hybrid exhibited CO and CH_4_ evolution rates of 41.2 and 13.7 μmol g⁻^1^, respectively, without any cocatalyst or sacrificial agent, which was 4 times higher than that of COF (Fig. 8b and c). However, a drawback of the CsPbBr_3_/COF system is its tendency to decompose in the presence of H_2_O, which not only reduces the stability of the heterostructure but also poses environmental risks due to the high toxicity of the decomposition products. To address these challenges, Li *et al*. [175] employed CdSe/CdS colloidal QDs instead of CsPbBr_3_ to prepare double-shelled TAPT-DMTA/NRs with a hierarchically porous structure (Fig. 8d). TEM images showed that CdSe/CdS nanorods self-assemble to form a loose inner core, which was then coated with a uniform bivalve hollow structure. These heterostructures feature layered pores that facilitate the separation of photoexcited electrons, resulting in a self-assembled porous nanocomposite that exhibits a higher CO generation rate (395 μmol g⁻^1^) under visible light irradiation compared to non-porous CdSe/CdS nanorods (Fig. 8e). They demonstrated that the produced CO originates from CO_2_ reduction through isotope experiments using labeled ^13^CO_2_ as a reactant (Fig. 8f). *In situ* diffuse reflectance infrared Fourier transform spectroscopy (DRIFTS) results revealed new peaks at 1284, 1510 and 1624 cm⁻^1^ (Fig. 8g), which were attributed to the vibration of COOH*, an intermediate in the reduction of CO_2_ to CO. The photocatalytic CO_2_ reduction of the COF-based heterojunction photocatalyst is summarized in Table 3.

**Table 3.** Summary of recent representative studies on COF-based heterojunction photocatalysts for CO_2_ reduction.

	Light source				
Compound	Filter (nm)	Power (W)	Activity (μmol g^−1^ h^−1^)	Selectivity (%)	Ref.
COF-318-TiO_2_	>300	300 (Xe)	CO, 278.7		[169]
BPDA-COF-TiO_2_	AM1.5	150 (Xe)	CO, 91		[170]
MoS_2_@COF	>420	300 (Xe)	C_2_H_6_, 56.2	83.8	[75]
UiO-66-NH_2_/COF-366-Co	>300	300 (Xe)	CO, 4092.2	73.3	[173]
M@C-Br-1	>320	300 (Xe)	CO, 106.4 CH_4_, 15.5	87.3 12.7	[174]
COF/QDs-II	>420	300 (Xe)	CO, 41.2 CH_4_, 13.7	75.1 24.9	[39]
TAPT-DMTA/NR40-H	>420	300 (Xe)	CO, 64.6		[175]
APTES-TiO_2_	>420	300 (Xe)	CO, 6.8	87.6	[69]
TiO_2_/Tp-Tta COF	>420	300 (Xe)	CO, 580	95	[73]
PRGO/TP-COF	>400	300 (Xe)	CO, 9.8		[58]
NH_2_-MIL-125@COF-3	AM 1.5	300 (Xe)	CO, 22.9 CH_4_, 4.0	85.1 14.9	[176]
CuS_x_/TP-TA	>420	300 (Xe)	CO, 6.8		[177]
CIS@TCOF	>420	300 (Xe)	CO, 35.2 COOH, 171.2	17.1 82.9	[110]
C_60_/TpPa		LED	CO, 48.2 (pure CO_2_) CO, 90.3 (10% CO_2_)		[61]
C_3_N_4_ (NH)/COF	>400	300 (Xe)	CO, 11.3	90.4	[64]
HBWO@Br-COF-2	AM 1.5	300 (Xe)	CO, 19.9 CH_4_, 0.65	96.8 3.2	[178]
WO_3_/THFB-COF-Zn	>420	300 (Xe)	CO, 51.4	0.6	[74]
Br-COFs@BiOCl	>420	300 (Xe)	CO, 27.4		[179]

### H_2_O_2_ production

H_2_O_2_ plays a crucial role in various fields, including bleaching, organic synthesis, energy storage and water purification [180–183]. Currently, H_2_O_2_ is mainly produced by the energy-intensive anthraquinone oxidation process, which poses safety risks caused by high temperatures and pressures, along with environmental concerns stemming from the release of toxic by-products [181]. As a result, there is a growing need to develop alternative production methods. Photocatalysis offers a promising solution by utilizing solar energy to convert earth-abundant O_2_ and H_2_O into H_2_O_2_ [184–190]. COFs have garnered considerable attention for their ability to promote separation by spatially isolating electrons and holes through the strategic design of monomer structures without requiring sacrificial agents to quench excitons. However, COFs are hindered by high exciton binding energies and limited stability. To overcome these challenges, constructing COF-based heterojunctions provides an effective strategy for efficient photocatalytic H_2_O_2_ production.

**Figure 9.**
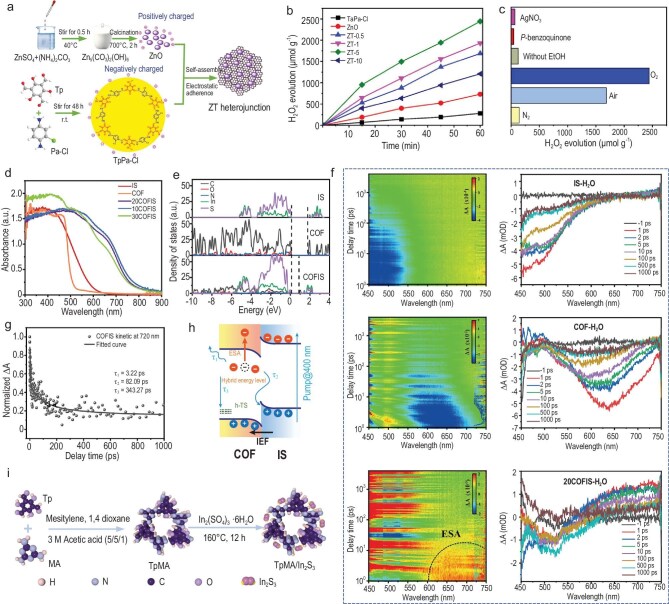
(a) The synthetic process for ZnO/TpPa-Cl heterostructures. (b) Time-dependent photocatalytic H_2_O_2_ production toward as-prepared photocatalysts in 10% ethanol/H_2_O under light irradiation. (c) Comparison of H_2_O_2_ production catalyzed by the ZnO/TpPa-Cl composite in different atmospheres (N_2_/O_2_/Air) and in the presence and absence of reactive species scavengers [70]; Copyright 2022, Elsevier. (d) The UV-vis DRS spectra of as-prepared samples. (e) Calculated band gaps of In_2_S_3_, COF and COFIS. (f) Fs-TA spectra of In_2_S_3_, COF and COFIS under 400 nm laser pulse excitation in H_2_O. (g) The normalized decay kinetic curves of 20COFIS at 720 nm. (h) Schematics of the charge transfer pathways of COFIS [67]; Copyright 2024, Wiley-VCH. (i) The synthetic process for TpMA/In_2_S_3_ heterostructures [191]; Copyright 2024, Elsevier.

Zhang *et al*. [70] integrated positively charged ZnO particles with negatively charged TpPa-Cl through electrostatic self-assembly (Fig. 9a). As shown in Fig. 9b, the optimal ZnO/TpPa-Cl composite achieved an H_2_O_2_ production rate of 2443 mmol g^−1^ h^−1^ in 10 vol% ethanol oxygen-saturated solution, which is 3.3- and 8.7-fold higher than that of ZnO (812 μmol g^−1^ h^−1^) and TpPa-Cl (281 μmol g^−1^ h^−1^). The enhanced performance was attributed to the formation of a dense and stable interface via electrostatic self-assembly, which effectively promoted interfacial charge separation. Besides, the photocatalytic activity was reduced in N_2_ atmosphere and O_2_-saturated ethanol/H_2_O containing *p*-benzoquinone, indicating that the conversion of O_2_→^•^O_2_^−^ was a crucial step during the process of H_2_O_2_ production (Fig. 9c). However, ethanol was used as a sacrificial agent to consume photogenerated holes and promote charge separation, which resulted in the ZnO-COF composite exhibiting higher performance of H_2_O_2_ production in 10% ethanol solution. Subsequently, they further synthesized COF/In_2_S_3_ S-scheme photocatalysts, achieving an impressive H_2_O_2_ yield of 5713.2 μmol g^−1^ h^−1^ without any sacrificial agent [67]. Notably, unlike other heterojunctions that typically absorb light within the range of their individual components, the COF/In_2_S_3_ composite (COFIS) exhibited a red shift in its light absorption (Fig. 9d). This red shift was attributed to the coordination of N in the COF with In^3^⁺, which promoted electron delocalization and the formation of hybrid energy levels, effectively reducing the band gap (Fig. 9e). In the fs-TA spectra of COFIS, a new positive absorption feature corresponding to excited state absorption (ESA) was observed (Fig. 9f), while it was absent in COF and In_2_S_3_. Therefore, the ESA signal corresponded to the transfer of photogenerated electrons from the hybrid energy levels in COFIS to the CB of COF. For COFIS, the ESA decay curve at 720 nm was fitted using a triple-exponential model, revealing three relaxation pathways: *τ*_1_ (3.22 ps) corresponding to electron diffusion, *τ*_3_ (343.27 ps) related to the recombination of electrons and holes (Fig. 9g), and the unique *τ*_2_ (82.09 ps) attributed to the recombination of photogenerated electrons in the hybrid energy levels with photogenerated holes in In_2_S_3_ (Fig. 9h). Compared to electron-hole recombination in pure COF (107.71 ps), the enhanced lifetime of photogenerated carriers indicated that a portion of photogenerated electrons in COF flowed in In_2_S_3_ via the S-scheme transfer mechanism. Bi and co-workers [191] modified the monomer structure of COF using 1,3,5-triformylphloroglucinol and melamine to synthesize similar COF/In_2_S_3_ S-scheme heterojunctions (Fig. 9i). Notably, they designed a ‘Photocatalysis-Fenton’ system by combining the produced H_2_O_2_ under light irradiation with Fe(II), demonstrating its bactericidal ability.

BiOBr is a typical 2D layered structure that is commonly used in the construction of BiOBr/COF heterostructures due to its suitable band structure and easy preparation [192]. Wang and co-workers successfully synthesized an S-scheme BiOBr/COF composite by growing BiOBr nanosheets *in situ* on the large π-conjugated skeleton of COF [49]. The BiOBr/COF composite (BT40) demonstrated the highest H_2_O_2_ yield of 3749 μmol g^−1^ h^−1^, which was 1.85 and 27 times greater than those of the COF and BiOBr. This enhanced activity was attributed to the relatively low decomposition rate constants and high formation rate constants of BT40. DFT calculations indicated that the adsorption energy for the O_2_ molecule in a lying-down configuration near the C=N in COF was −0.64 eV, significantly lower than −0.24 eV for the standing-up configuration. Furthermore, the electron transfer numbers for O_2_ under these two adsorption configurations were 0.3 and 0.24, respectively. These results suggest that O_2_ is more likely to be adsorbed in the lying-down configuration. Notably, this O_2_ adsorption configuration favored a coordinated two-electron reduction pathway, as the two O atoms exhibited similar reducibility and accessibility, allowing for the reduction and protonation of both O atoms to form H_2_O_2_ in a single step. Recently, singlet oxygen (^1^O_2_) has been shown to play a crucial role in photocatalytic H_2_O_2_ production within COF systems. For example, Jiang *et al*. developed a hollow TiO_2_@TpPa (TOTP) S-scheme heterojunction through Schiff base reactions between 1,3,5-triformylphloroglucinol and paraphenylenediamine [193]. The optimized TOTP2.4 composite achieved an H_2_O_2_ production rate of 891 μmol g^−1^ h^−1^ in pure water. Mechanistic studies revealed that rapid phototautomerization of the COF from enol to keto configuration enhanced π-electron delocalization, thereby promoting ^1^O_2_ generation. Time-resolved EPR spectroscopy directly confirmed ^1^O_2_ formation, while complementary DFT calculations and *in situ* spectroscopic analyses elucidated the efficient charge separation in this S-scheme system. This unique ^1^O_2_-mediated pathway enables more efficient H_2_O_2_ production by avoiding a large spin-forbidden barrier.

### Organic synthesis

Photocatalytic organic synthesis has emerged as a transformative approach in green chemistry, providing a sustainable alternative to conventional synthetic methods [194–197]. By harnessing light energy, photocatalysis enables various organic transformations under mild conditions, reducing the reliance on harsh reagents and excessive energy inputs. This method facilitates the activation of stable substrates, such as alkenes, alcohols, and aromatic compounds, to undergo selective processes, including functionalization, polymerization, and cross-coupling [198]. COF-based heterojunctions have garnered considerable attention due to their high affinity for organic substrates and the versatility afforded by tuned band engineering.

Recently, Wang *et al*. [85] reported a nanoscale 0D MAPbBr_3_/1D COF S-scheme heterostructure (MAPB-T-COF) via a straightforward *in situ* growth-assembly method. MAPB-T-COF exhibited high photocatalytic performance in converting 4-methylbenzenethiol to *p*-tolyl disulfide under blue LED irradiation. Specifically, the composite achieved a 100% yield and the AQY was 12.76%. They claimed that MAPbBr_3_ served as the active site of catalytic coupling reaction driven by blue light. In this process, the –SH group was attacked by ^•^O_2_⁻ radicals and lost a proton, accepting a hole from the VB of MAPbBr_3_, leading to the formation of –S^•^ radicals. The coupling of two –S^•^ radicals subsequently resulted in the formation of the S–S bond. Using a continuous growth strategy, the team further synthesized a Ti-MOF@TpTt hybrid material coated with ultra-thin COF nanobelts [26]. This approach provided more anchoring sites for COF on Ti-MOF, generating a fibrous structure distinct from bulk COF. By controlling the amount of ligands during TpTt COF synthesis, the COF grew vertically on the Ti-MOF surface, forming ultra-thin nanobelt structures. Pd nanoparticles loaded *in situ* on the Ti-MOF@TpTt composite demonstrated good catalytic performance in ammonia borane hydrolysis and nitroarene hydrogenation. The high photocatalytic performance of the composite was attributed to (1) the formation of a type II heterojunction between Ti-MOF and TpTt COF, which enhanced the separation of photogenerated charge carriers; (2) the core-shell structure, which further promoted the spatial separation of charge carriers; and (3) the ultra-thin COF nanobelt shell, loaded with Pd NPs, increasing the exposure of active sites for photocatalytic reaction.

Integrating hydrogen production with organic synthesis within a single system holds great potential for achieving dual-functional value-added transformations. Han *et al*. [155] developed a multi-functional photocatalyst, Cu-NH_2_-MIL-125/TpPa-2-COF, which enables hydrogen production coupled with the efficient oxidation of benzylamine. The covalent bonding of the heterojunction enhances the separation of photogenerated charges, while the monodispersed transient Cu^2+^/Cu^+^ centers serve as highly effective active sites, greatly improving photocatalytic performance. The Cu-NH_2_-MIL-125/TpPa-2-COF (4:6) catalyst achieved a benzylamine oxidation conversion rate of 91.2% with a selectivity >99%. Additionally, it demonstrated a hydrogen production rate of 9.21 mmol g^−1^ h^−1^, which is 7.3 and 17.7 times higher than the Cu-free and pure TpPa-2-COF photocatalysts, respectively.

### Other applications

COF-based heterostructures have also been investigated for other applications, including U(VI) and Cr (VI) reduction [199], sterilization [200] and N_2_ fixation [201,202], which is briefly reviewed below.

The tuned active sites and ordered pores of COF, composed of diverse structural units, make them excellent carriers for uranium adsorption and diffusion. Liu *et al*. [199] synthesized a dendritic COF using 2,4,6-triformylphloroglucinol and 1,3,5-triazine-2,4,6-triamine, followed by the *in situ* growth of SnS_2_ particles on its surface. Their study revealed that various anions had minimal impact on U(VI) removal, primarily due to the strong binding energy between the hybrid material and U(VI). Even when the concentration of competing cations was five times higher than that of U(VI), the reduction rate of U(VI) remained above 95%.

Upon illumination, highly oxidative free radicals are generated within the COF-based heterojunction, decomposing microbial metabolites and intracellular substances, and resulting in complete sterilization. A core-shell COF@Co_3_O_4_ Z-scheme heterostructure with extensive interfacial contact was developed and utilized as both a nanocatalyst and sonosensitizers for synergistic nanocatalytic-sonodynamic tumor therapy [200]. The composite demonstrated enhanced production of reactive oxygen species and enzyme-mimicking catalytic activity, attributed to the improved charge separation in the Z-scheme heterojunction. The COF@Co_3_O_4_ composite achieved ^1^O_2_ and ^•^OH generation efficiencies that were 2.3 and 2.1 times higher, respectively, than those of pure Co_3_O_4_.

There are relatively fewer reports on nitrogen fixation using COF-based heterojunctions because it is difficult to activate the N_2_ molecules. Wang *et al*. [28] developed a CdS/TpPa-1 heterojunction with a tight contact interface by adsorbing Cd^2+^ and *in situ* growing of CdS on TpPa-1. The optimized composite achieved a maximum photocatalytic NH_3_ production rate of 241 μmol g^−1^ h^−1^ without the use of any cocatalysts or sacrificial agents, which was 3.0 times and 1.7 times higher than that of CdS and TpPa-1, respectively. Under N_2_ atmosphere, the peak at 3400–3560 nm detected by *in situ* DRIFTS was intensified over time, reflecting the stretching vibrations associated with the generated NH_3_. Isotope labeling experiments further confirmed that the nitrogen in the NH_3_ originated from N_2_ gas.

## CHALLENGES AND OUTLOOK

In summary, COF-based heterojunction photocatalysts offer substantial prospects for solar-to-chemical conversion due to their long-range ordered structure, tunable porosity, large specific surface area and efficient charge separation. This review has explored the mechanisms, classifications, and synthetic strategies of COF-based heterostructure photocatalysts. The tailored design of monomer molecules allows for the precise tuning of properties such as porosity and catalytic activity to satisfy the specific functions of COF-based heterostructures. Additionally, the latest advancements in COF-based heterostructures within the field of photocatalysis are summarized, covering applications such as pollutant photodegradation, photocatalytic H_2_ evolution, CO_2_ reduction, H_2_O_2_ production and organic synthesis. The synergistic effects of COF and other supporting materials in relation to structure–activity relationships are highlighted.

**Figure 10.**
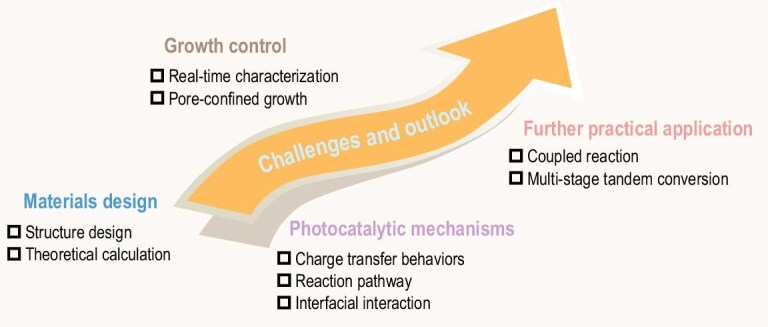
Challenges and outlook of COF-based heterojunctions.

Although the construction of COF-based heterojunctions has improved carrier separation efficiency, their development is still at the early stage. Creating efficient and durable COF-based heterojunction photocatalysts remains challenging (Fig. 10):

1. It is essential to design materials that address the instability of heterojunctions under certain reaction conditions, such as extreme pH or high temperatures, and prolonged photocatalytic operations to ensure their long-term high activity. COFs provide structural design flexibility, and when combined with other materials, these strategies can promote their stability and photocatalytic efficiency. Theoretical calculations play a key role in optimizing contact interfaces, reducing experimental workload, and predicting the stability of these hybrid materials. The combination of computational modeling and structure design is expected to pave the way for the creation of more stable and efficient photocatalytic systems. Besides, crucial considerations for the design of COF-based photocatalysts for large-scale applications also include scalability and economic feasibility. The design process should focus on scalable synthesis methods while optimizing the cost of materials synthesis to ensure the economic viability, competitiveness, and sustainability of COF-based photocatalysts.
2. Achieving precise control over the growth of hybrid photocatalysts. A key challenge in the synthesis of hybrid materials is the uniformity of the heterojunctions, especially when COFs, which are typically composed of micrometer-sized bulk stacks, are involved. Variability in the thickness and composition of these structures leads to inconsistent catalytic performance. Therefore, it is essential to achieve precise control over the growth of these materials, potentially by using the inherent pores in COFs to confine growth. Furthermore, real-time monitoring techniques, such as *in situ* TEM and X-ray diffraction, can provide valuable insights into growth dynamics, enabling the precise tailoring of material morphology and composition to optimize their photocatalytic performance.
3. Understanding the fundamental processes of photocatalysis in organic-inorganic hybrids. An in-depth molecular understanding of the fundamental processes of photocatalysis in organic-inorganic heterostructures has not yet been achieved. Understanding the internal mechanisms of these heterojunctions is essential for elucidating the structure–activity relationship. By examining factors such as functional group arrangement, pore size, and interfacial interactions that affect charge carrier dynamics and photocatalytic efficiency, key design parameters for optimization to achieve efficient charge transfer can be identified. Advanced techniques like *in situ* spectroscopy and computational models, will offer insights into charge transfer kinetics and reaction pathways.
4. It is important to explore the value-added chemical synthesis. The development of heterojunction photocatalytic materials enables spatial separation and directional migration of photogenerated charge carriers while preserving useful electrons and holes for redox reactions. This approach facilitates the coupling of photocatalytic reduction with organic oxidation reactions, enhancing atomic economy and solar-to-chemical conversion efficiency. From the point of view of practical applications, the great promise of photocatalytic reduction coupled with organic upgrading such as the synthesis of pharmaceutical intermediates, gas synthesis, and liquid fuels etc., shows promise, but this field is still at an early stage of development. Several challenges, including the limited variety of reaction types, low catalytic activity, and poor selectivity toward target products, remain to be resolved. Integrating both oxidation and reduction active sites enhances the catalytic efficiency and selectivity, enabling more complex reactions and facilitating the effective conversion of a broader range of substrates. A key aspect is the *in situ* utilization of reduction products and organic substrates. For example, the development of multi-stage tandem conversion systems for the transformation of CO_2_ into value-added products could offer substantial economic and environmental benefits.

## References

[1] Ma J, Miao TJ, Tang J. Charge carrier dynamics and reaction intermediates in heterogeneous photocatalysis by time-resolved spectroscopies. Chem Soc Rev 2022; 51: 5777–94. 10.1039/D1CS01164B 35770623

[2] Bie C, Wang L, Yu J. Challenges for photocatalytic overall water splitting. Chem 2022; 8: 1567–74. 10.1016/j.chempr.2022.04.013

[3] Nikoloudakis E, Lopez-Duarte I, Charalambidis G et al. Porphyrins and phthalocyanines as biomimetic tools for photocatalytic H_2_ production and CO_2_ reduction. Chem Soc Rev 2022; 51: 6965–7045. 10.1039/D2CS00183G 35686606

[4] Kondo Y, Kuwahara Y, Mori K et al. Design of metal-organic framework catalysts for photocatalytic hydrogen peroxide production. Chem 2022; 8: 2924–38. 10.1016/j.chempr.2022.10.007

[5] Zheng K, Wu Y, Hu Z et al. Progress and perspective for conversion of plastic wastes into valuable chemicals. Chem Soc Rev 2023; 52: 8–29. 10.1039/D2CS00688J 36468343

[6] Su B-L . Photocatalytic hydrogen production toward carbon neutrality: tracking charge separation. Natl Sci Rev 2023; 10: nwad139. 10.1093/nsr/nwad139 37565209 PMC10411669

[7] Yang C, Cheng B, Xu J et al. Donor-acceptor-based conjugated polymers for photocatalytic energy conversion. EnergyChem 2024; 6: 100116. 10.1016/j.enchem.2023.100116

[8] Xiang Q, Yu J, Jaroniec M. Graphene-based semiconductor photocatalysts. Chem Soc Rev 2012; 41: 782–96. 10.1039/C1CS15172J 21853184

[9] Ran J, Zhang J, Yu J et al. Earth-abundant cocatalysts for semiconductor-based photocatalytic water splitting. Chem Soc Rev 2014; 43: 7787–812. 10.1039/C3CS60425J 24429542

[10] Wang Y, Hu J, Yu L et al. Recent strategies for constructing efficient interfacial solar evaporation systems. Nano Res Energy 2023; 2: e9120062. 10.26599/NRE.2023.9120062

[11] He F, Weon S, Jeon W et al. Self-wetting triphase photocatalysis for effective and selective removal of hydrophilic volatile organic compounds in air. Nat Commun 2021; 12: 6259. 10.1038/s41467-021-26541-z 34716347 PMC8556241

[12] Zhang J, Yu Z, Gao Z et al. Porous TiO_2_ nanotubes with spatially separated platinum and CoO_x_ cocatalysts produced by atomic layer deposition for photocatalytic hydrogen production. Angew Chem Int Ed 2017; 56: 816–20. 10.1002/anie.201611137 27966808

[13] Lin C-Y, Lai Y-H, Mersch D et al. Cu_2_O|NiO*x* nanocomposite as an inexpensive photocathode in photoelectrochemical water splitting. Chem Sci 2012; 3: 3482–7. 10.1039/c2sc20874a

[14] Bhattacharyya S, Ehrat F, Urban P et al. Effect of nitrogen atom positioning on the trade-off between emissive and photocatalytic properties of carbon dots. Nat Commun 2017; 8: 1401. 10.1038/s41467-017-01463-x 29123091 PMC5680170

[15] Cheng C, He B, Fan J et al. An inorganic/organic S-scheme heterojunction H_2_-production photocatalyst and its charge transfer mechanism. Adv Mater 2021; 33: 2100317. 10.1002/adma.202100317 33904199

[16] Li Y, Wan S, Liang W et al. D-A conjugated polymer/CdS S-scheme heterojunction with enhanced interfacial charge transfer for efficient photocatalytic hydrogen generation. Small 2024; 20: 2312104. 10.1002/smll.202312104 38441363

[17] Peng H, Liu D, Zheng X et al. N-doped carbon-coated ZnS with sulfur-vacancy defect for enhanced photocatalytic activity in the visible light region. Nanomaterials 2019; 9: 1657. 10.3390/nano9121657 31766440 PMC6956101

[18] Li H, Sun S, Ji H et al. Enhanced activation of molecular oxygen and degradation of tetracycline over Cu-S_4_ atomic clusters. Appl Catal B 2020; 272: 118966. 10.1016/j.apcatb.2020.118966

[19] Chen R, Wang Y, Ma Y et al. Rational design of isostructural 2D porphyrin-based covalent organic frameworks for tunable photocatalytic hydrogen evolution. Nat Commun 2021; 12: 1354. 10.1038/s41467-021-21527-3 33649344 PMC7921403

[20] Zhang L, Zhang J, Yu J et al. Charge-transfer dynamics in S-scheme photocatalyst. Nat Rev Chem 2025; 9: 328–42. 10.1038/s41570-025-00698-3 40097789

[21] Cheng J, Cheng B, Xu J et al. Organic-inorganic S-scheme heterojunction photocatalysts: design, synthesis, applications, and challenges. eScience 2025; 5: 100354. 10.1016/j.esci.2024.100354

[22] Diao X, Zhang X, Li Y et al. Heterogeneous network of 2D MOFs decorated 1D CNTs imparting multiple functionalities to composite phase change materials. Nano Res Energy 2024; 3: e9120114. 10.26599/NRE.2024.9120114

[23] Zhang L, Wang Y, Chu A et al. Facet-selective growth of halide perovskite/2D semiconductor van der Waals heterostructures for improved optical gain and lasing. Nat Commun 2024; 15: 5484. 10.1038/s41467-024-49364-0 38942769 PMC11213932

[24] Luo Y, Wang X, Wang P et al. Inorganic/organic hybrid interfacial internal electric field modulated charge separation of resorcinol-formaldehyde resin for boosting photocatalytic H_2_O_2_ production. Chem Eng J 2024; 497: 154886. 10.1016/j.cej.2024.154886

[25] Zhou Y, Wang Z, Yang P et al. Electronic and optical properties of two-dimensional covalent organic frameworks. J Mater Chem 2012; 22: 16964–70. 10.1039/c2jm32321d

[26] Zhang M-Y, Li J-K, Wang R et al. Construction of core-shell MOF@COF hybrids with controllable morphology adjustment of COF shell as a novel platform for photocatalytic cascade reactions. Adv Sci 2021; 8: 2101884. 10.1002/advs.202101884 PMC8498909 34378352

[27] Xu N, Liu Y, Yang W et al. 2D-2D heterojunctions of a covalent triazine framework with a triphenylphosphine-based covalent organic framework for efficient photocatalytic hydrogen evolution. ACS Appl Energy Mater 2020; 3: 11939–46. 10.1021/acsaem.0c02102

[28] Wang C, Shi S, Duan F et al. Anchoring ultrafine CdS nanoparticles in TpPa-1-COF: a type II heterojunction for enhanced photocatalytic N_2_ fixation. J Mater Chem A 2022; 10: 16524–32. 10.1039/D2TA03461A

[29] Haase F, Lotsch BV. Solving the COF trilemma: towards crystalline, stable and functional covalent organic frameworks. Chem Soc Rev 2020; 49: 8469–500. 10.1039/D0CS01027H 33155009

[30] Sun L, Lu M, Yang Z et al. Nickel glyoximate based metal-covalent organic frameworks for efficient photocatalytic hydrogen evolution. Angew Chem Int Ed 2022; 61: e202204326. 10.1002/anie.202204326 35561154

[31] Jin Y, Zhi Q, Wang H et al. Robust dioxin-linked metallophthalocyanine tbo topology covalent organic frameworks and their photocatalytic properties. Natl Sci Rev 2024; 12: nwae396. 10.1093/nsr/nwae396 39831002 PMC11740510

[32] Xu Y, Xue H, Li X et al. Application of metal-organic frameworks, covalent organic frameworks and their derivates for the metal-air batteries. Nano Res Energy 2023; 2: e9120052. 10.26599/NRE.2023.9120052

[33] Gong Y, Guan X, Jiang H. Covalent organic frameworks for photocatalysis: synthesis, structural features, fundamentals and performance. Coord Chem Rev 2023; 475: 214889. 10.1016/j.ccr.2022.214889

[34] Wang Q, Liu J, Cao M et al. Aminal-linked porphyrinic covalent organic framework for rapid photocatalytic decontamination of mustard-gas simulant. Angew Chem Int Ed 2022; 61: e202207130. 10.1002/anie.202207130 35672265

[35] Qu J, Wang Y, Sun T et al. Engineering covalent organic frameworks for photocatalytic overall water vapor splitting. Angew Chem Int Ed 2025; 64: e202502821. 10.1002/anie.202502821 40125712

[36] Li T, Zhang P-L, Dong L-Z et al. Post-synthetic rhodium (III) complexes in covalent organic frameworks for photothermal heterogeneous C-H activation. Angew Chem Int Ed 2024; 63: e202318180. 10.1002/anie.202318180 38242848

[37] Li Q, Li X, Zhang B et al. Recent advancements in COFs-based heterostructures for CO_2_ photoreduction. Adv Funct Materials 2025; 35: 2506421. 10.1002/adfm.202506421

[38] Geng K, He T, Liu R et al. Covalent organic frameworks: design, synthesis, and functions. Chem Rev 2020; 120: 8814–933. 10.1021/acs.chemrev.9b00550 31967791

[39] He Y, Hu P, Zhang J et al. Boosting artificial photosynthesis: CO_2_ chemisorption and S-scheme charge separation via anchoring inorganic QDs on COFs. ACS Catal 2024; 14: 1951–61. 10.1021/acscatal.4c00026

[40] Wang W, Cheng B, Luo G et al. S-scheme heterojunction photocatalysts based on 2D materials. Mater Today 2024; 81: 137–58. 10.1016/j.mattod.2024.10.006

[41] Li T, Tsubaki N, Jin Z. S-scheme heterojunction in photocatalytic hydrogen production. J Mater Sci Technol 2024; 169: 82–104. 10.1016/j.jmst.2023.04.049

[42] Côté AP, Benin AI, Ockwig NW et al. Porous, crystalline, covalent organic frameworks. Science 2005; 310: 1166–70. 16293756 10.1126/science.1120411

[43] Zhang Y, Hu Z, Zhang H et al. Uncovering original Z scheme heterojunctions of COF/MO_x_ (M = Ti, Zn, Zr, Sn, Ce, and Nb) with ascendant photocatalytic selectivity for virtually 99.9% NO-to-NO_3_^−^ oxidation. Adv Funct Mater 2023; 33: 2303851. 10.1002/adfm.202303851

[44] Bao S, Tan Q, Wang S et al. TpBD COF@ZnIn_2_S_4_ nanosheets: a novel S-scheme heterojunction with enhanced photoreactivity for hydrogen production. Appl Catal B 2023; 330: 122624. 10.1016/j.apcatb.2023.122624

[45] Shang D, Li D, Chen B et al. 2D-2D SnS_2_/covalent organic framework heterojunction photocatalysts for highly enhanced solar-driven hydrogen evolution without cocatalysts. ACS Sustain Chem Eng 2021; 9: 14238–48. 10.1021/acssuschemeng.1c05162

[46] Yang D, Li Z-G, Zhang X et al. Rational design of ZnCdS/TpPa-1-COF heterostructure photocatalyst by strengthening the interface connection in solar hydrogen production reactions. Nano Res 2024; 17: 1027–34. 10.1007/s12274-023-5991-5

[47] Zhong X, Liu Y, Wang S et al. In-situ growth of COF on BiOBr 2D material with excellent visible-light-responsive activity for U(VI) photocatalytic reduction. Sep Purif Technol 2021; 279: 119627. 10.1016/j.seppur.2021.119627

[48] Zhang Y, Chen Z, Shi Z et al. A direct Z-scheme BiOBr/TzDa COF heterojunction photocatalyst with enhanced performance on visible-light driven removal of organic dye and Cr(VI). Sep Purif Technol 2021; 275: 119216. 10.1016/j.seppur.2021.119216

[49] Zhang H, Liu J, Zhang Y et al. BiOBr/COF S-scheme photocatalyst for H_2_O_2_ production via concerted two-electron pathway. J Mater Sci Technol 2023; 166: 241–9. 10.1016/j.jmst.2023.05.030

[50] Guo J, Ma D, Sun F et al. Substituent engineering in g-C_3_N_4_/COF heterojunctions for rapid charge separation and high photo-redox activity. Sci China Chem 2022; 65: 1704–9. 10.1007/s11426-022-1350-1

[51] Lu W, Qin T, Wang B et al. Construction of α-Fe_2_O_3_/g-C_3_N_4_/COF ternary hybrid with double Z-scheme heterojunctions for photocatalysis. Chemphotochem 2023; 7: e202200230. 10.1002/cptc.202200230

[52] Jiang J, Zhou S, Chen Z et al. Facile fabrication of a visible-light stable metal-free g-C_3_N_4_/COF heterojunction with efficiently enhanced photocatalytic activity. New J Chem 2023; 47: 7538–47. 10.1039/D2NJ05532E

[53] Zhang L, Xi T, Zhu D et al. Adsorption-enhanced photocatalytic waterborne virus inactivation by graphite carbon nitride conjugated with covalent organic frameworks. Chem Eng J 2023; 472: 144893. 10.1016/j.cej.2023.144893

[54] He S, Rong Q, Niu H et al. Platform for molecular-material dual regulation: a direct Z-scheme MOF/COF heterojunction with enhanced visible-light photocatalytic activity. Appl Catal B 2019; 247: 49–56. 10.1016/j.apcatb.2019.01.078

[55] Wan Y, Yang H, Shang Q et al. Integrating hollow spherical covalent organic frameworks on NH_2_-MIL-101(Fe) as high performance heterogeneous photocatalysts. Environ Sci: Nano 2022; 9: 3081–93. 10.1039/D2EN00393G

[56] Hou L, Gao Y, Kong F et al. Reticular heterojunction for organic photoelectrochemical transistor detection of neuron-specific enolase. Small 2024; 20: 2400033. 10.1002/smll.202400033 38431941

[57] Deng M, Guo J, Ma X et al. Enhanced photocatalytic Cr(VI) reduction performance by novel PDI/COFs composite. Sep Purif Technol 2023; 326: 124786. 10.1016/j.seppur.2023.124786

[58] Liu Y, Wang Y, Shang J et al. Construction of a novel metal-free heterostructure photocatalyst PRGO/TP-COF for enhanced photocatalytic CO_2_ reduction. Appl Catal B 2024; 350: 123937. 10.1016/j.apcatb.2024.123937

[59] Ou S, Zhou M, Chen W et al. COF-5/CoAl-LDH nanocomposite heterojunction for enhanced visible-light-driven CO_2_ reduction. ChemSusChem 2022; 15: e202200184. 10.1002/cssc.202200184 35187792

[60] Wang W, Wang W, Liang Y et al. Advanced stimuli-responsive structure based on 4D aerogel and covalent organic frameworks composite for rapid reduction in tetracycline pollution. Molecules 2023; 28: 5505. 10.3390/molecules28145505 37513377 PMC10386521

[61] He Y, Fu Y, Meng X et al. Enhanced visible light-driven CO_2_ reduction activity induced by Z-scheme heterojunction photocatalyst C_60_/TpPa (COF). Appl Catal A 2023; 663: 119320. 10.1016/j.apcata.2023.119320

[62] Li F, Wang D, Xing Q-J et al. Design and syntheses of MOF/COF hybrid materials via postsynthetic covalent modification: an efficient strategy to boost the visible-light-driven photocatalytic performance. Appl Catal B 2019; 243: 621–8. 10.1016/j.apcatb.2018.10.043

[63] Zhang Y, Tang H, Dong H et al. Covalent-organic framework based Z-scheme heterostructured noble-metal-free photocatalysts for visible-light-driven hydrogen evolution. J Mater Chem A 2020; 8: 4334–40. 10.1039/C9TA12870K

[64] Wang J, Yu Y, Cui J et al. Defective g-C_3_N_4_/covalent organic framework van der Waals heterojunction toward highly efficient S-scheme CO_2_ photoreduction. Appl Catal B 2022; 301: 120814. 10.1016/j.apcatb.2021.120814

[65] Liang Z, Shen R, Zhang P et al. All-organic covalent organic frameworks/perylene diimide urea polymer S-scheme photocatalyst for boosted H_2_ generation. Chin J Catal 2022; 43: 2581–91. 10.1016/S1872-2067(22)64130-5

[66] Gao Z, Jian Y, Yang S et al. Interfacial Ti-S bond modulated S-scheme MOF/covalent triazine framework nanosheet heterojunctions for photocatalytic C-H functionalization. Angew Chem Int Ed 2023; 62: e202304173. 10.1002/anie.202304173 37132083

[67] Qiu J, Meng K, Zhang Y et al. COF/In_2_S_3_ S-scheme photocatalyst with enhanced light absorption and H_2_O_2_-production activity and fs-TA investigation. Adv Mater 2024; 36: 2400288. 10.1002/adma.202400288 38411357

[68] Zhou Y, Dong P, Liu J et al. Functional groups-dependent Tp-based COF/MgIn_2_S_4_ S-scheme heterojunction for photocatalytic hydrogen evolution. Adv Funct Mater 2025; 35: 2500733. 10.1002/adfm.202500733

[69] Wang L, Cheng H, Zhang Z et al. Rational design of honeycomb-like APTES-TiO_2_/COF heterostructures: promoted intramolecular charge transfer for visible-light-driven catalytic CO_2_ reduction. Chem Eng J 2023; 456: 140990. 10.1016/j.cej.2022.140990

[70] Zhang Y, Qiu J, Zhu B et al. ZnO/COF S-scheme heterojunction for improved photocatalytic H_2_O_2_ production performance. Chem Eng J 2022; 444: 136584. 10.1016/j.cej.2022.136584

[71] Li S, Yu H, Wang Y et al. Engineering covalently integrated COF@CeO_2_ Z-scheme heterostructure for visible light driven photocatalytic CO_2_ conversion. Appl Surf Sci 2023; 615: 156335. 10.1016/j.apsusc.2023.156335

[72] Yang Y, Liu J, Gu M et al. Bifunctional TiO_2_/COF S-scheme photocatalyst with enhanced H_2_O_2_ production and furoic acid synthesis mechanism. Appl Catal B 2023; 333: 122780. 10.1016/j.apcatb.2023.122780

[73] An X, Bian J, Zhu K et al. Facet-dependent activity of TiO_2_/covalent organic framework S-scheme heterostructures for CO_2_ photoreduction. Chem Eng J 2022; 442: 135279. 10.1016/j.cej.2022.135279

[74] Fang L, Bai L, Wu D et al. S-scheme heterojunction of WO_3_/metal-covalent organic frameworks for artificial photosynthetic CO_2_ reduction. Chem Eng J 2025; 510: 161820. 10.1016/j.cej.2025.161820

[75] Yang X, Lan X, Zhang Y et al. Rational design of MoS_2_@COF hybrid composites promoting C-C coupling for photocatalytic CO_2_ reduction to ethane. Appl Catal B 2023; 325: 122393. 10.1016/j.apcatb.2023.122393

[76] Dong P, Cheng T, Zhang J et al. Fabrication of an organic/inorganic hybrid TpPa-1-COF/ZnIn_2_S_4_ S-scheme heterojunction for boosted photocatalytic hydrogen production. ACS Appl Energy Mater 2023; 6: 1103–15. 10.1021/acsaem.2c03806

[77] Li H, Tao S, Wan S et al. S-scheme heterojunction of ZnCdS nanospheres and dibenzothiophene modified graphite carbon nitride for enhanced H_2_ production. Chin J Catal 2023; 46: 167–76. 10.1016/S1872-2067(22)64201-3

[78] Feng Y, Li J, Ye S et al. Growing COFs in situ on CdS nanorods as core-shell heterojunctions to improve the charge separation efficiency. Sustainable Energy Fuels 2022; 6: 5089–99. 10.1039/D2SE01159J

[79] Wang C, Wang L, Jin J et al. Probing effective photocorrosion inhibition and highly improved photocatalytic hydrogen production on monodisperse PANI@CdS core-shell nanospheres. Appl Catal B 2016; 188: 351–9. 10.1016/j.apcatb.2016.02.017

[80] Zhang Y, Zhou W, Jia L et al. Visible light driven hydrogen evolution using external and confined CdS: effect of chitosan on carriers separation. Appl Catal B 2020; 277: 119152. 10.1016/j.apcatb.2020.119152

[81] Yu K, He P, He N et al. CdS/COF core-shell nanorods with efficient chemisorption, enhanced carrier separation, and antiphotocorrosion ability for U(VI) photoreduction. Sci China Mater 2023; 66: 4680–8. 10.1007/s40843-023-2599-9

[82] Cui C, Xu X, Zhao X et al. Donor-acceptor covalent organic framework/ZnIn_2_S_4_ core-shell structure S-scheme heterostructure for efficient photocatalytic hydrogen evolution. Nano Energy 2024; 126: 109632. 10.1016/j.nanoen.2024.109632

[83] Liu F, Nie C, Dong Q et al. AgI modified covalent organic frameworks for effective bacterial disinfection and organic pollutant degradation under visible light irradiation. J Hazard Mater 2020; 398: 122865. 10.1016/j.jhazmat.2020.122865 32470769

[84] Shi Z, Chen Z, Zhang Y et al. COF TzDa/Ag/AgBr Z-scheme heterojunction photocatalyst for efficient visible light driven elimination of antibiotics tetracycline and heavy metal ion Cr(VI). Sep Purif Technol 2022; 288: 120717. 10.1016/j.seppur.2022.120717

[85] Wang Y, Li H, Lin Q et al. Nanoscale 0D/1D heterojunction of MAPbBr_3_/COF toward efficient LED-driven S-S coupling reactions. ACS Catal 2023; 13: 15493–504. 10.1021/acscatal.3c03051

[86] Lin Q, Tan S, Zhao J et al. Tunable band engineering management on perovskite MAPbBr_3_/COFs nano-heterostructures for efficient S-S coupling reactions. Small 2024; 20: 2304776. 10.1002/smll.202304776 37658502

[87] Hou Y, Cui C, Zhang E et al. A hybrid of g-C_3_N_4_ and porphyrin-based covalent organic frameworks via liquid-assisted grinding for enhanced visible-light-driven photoactivity. Dalton Trans 2019; 48: 14989–95. 10.1039/C9DT03307F 31498343

[88] Xing Y, Yin L, Zhao Y et al. Construction of the 1D covalent organic framework/2D g-C_3_N_4_ heterojunction with high apparent quantum efficiency at 500 nm. ACS Appl Mater Interfaces 2020; 12: 51555–62. 10.1021/acsami.0c15780 33156604

[89] Hassan AE, Elewa AM, Hussien MSA et al. Designing of covalent organic framework/2D g-C_3_N_4_ heterostructure using a simple method for enhanced photocatalytic hydrogen production. J Colloid Interface Sci 2024; 653: 1650–61. 10.1016/j.jcis.2023.10.010 37812841

[90] Xiao Z, Yusuf A, Ren Y et al. High-efficiency NO conversion via in-situ grown covalent organic framework on g-C_3_N_4_ nanosheets with single-atom platinum photocatalyst. Chem Eng J 2024; 497: 154487. 10.1016/j.cej.2024.154487

[91] Ding Y, Chen Y, Zhang X et al. Controlled intercalation and chemical exfoliation of layered metal-organic frameworks using a chemically labile intercalating agent. J Am Chem Soc 2017; 139: 9136–9. 10.1021/jacs.7b04829 28651432

[92] Deng X, Yang L, Huang H et al. Shape-defined hollow structural Co-MOF-74 and metal nanoparticles@Co-MOF-74 composite through a transformation strategy for enhanced photocatalysis performance. Small 2019; 15: 1902287. 10.1002/smll.201902287 31304675

[93] Tian J, Lv W, Shen A et al. Construction of the copper metal-organic framework (MOF)-on-indium MOF Z-scheme heterojunction for efficiently photocatalytic reduction of Cr (VI). Sep Purif Technol 2023; 327: 124903. 10.1016/j.seppur.2023.124903

[94] Sun K, Qian Y, Li D et al. Reticular materials for photocatalysis. Adv Mater 2024; 2411118. 10.1002/adma.202411118 39601158

[95] Niu Q, Dong S, Tian J et al. Rational design of novel COF/MOF S-scheme heterojunction photocatalyst for boosting CO_2_ reduction at gas-solid interface. ACS Appl Mater Interfaces 2022; 14: 24299–308. 10.1021/acsami.2c02439 35593448

[96] Ahmadijokani F, Ghaffarkhah A, Molavi H et al. COF and MOF hybrids: advanced materials for wastewater treatment. Adv Funct Mater 2024; 34: 2305527. 10.1002/adfm.202305527

[97] Wang J, Feng Y, Zhang B. MOF-COF hybrid frameworks materials. Prog Chem 2022; 34: 1308–20.

[98] Hu H, Zhang X, Zhang K et al. Construction of a 2D/2D crystalline porous materials based S-scheme heterojunction for efficient photocatalytic H_2_ production. Adv Energy Mater 2024; 14: 2303638. 10.1002/aenm.202303638

[99] Chu X, Liu S, Luan B-B et al. Crystal-facet-controlled internal electric field in MOF/COF heterojunction towards efficient photocatalytic overall water splitting. Angew Chem Int Ed 2025; 64: e202422940. 10.1002/anie.202422940 39976153

[100] Peng Y, Zhao M, Chen B et al. Hybridization of MOFs and COFs: a new strategy for construction of MOF@COF core-shell hybrid materials. Adv Mater 2018; 30: 1705454. 10.1002/adma.201705454 29134677

[101] Lv S-W, Liu J-M, Yang F-E et al. A novel photocatalytic platform based on the newly-constructed ternary composites with a double p-n heterojunction for contaminants degradation and bacteria inactivation. Chem Eng J 2021; 409: 128269. 10.1016/j.cej.2020.128269

[102] Yang Y, Zhao W, Niu H et al. Mechanochemical construction 2D/2D covalent organic nanosheets heterojunctions based on substoichiometric covalent organic frameworks. ACS Appl Mater Interfaces 2021; 13: 42035–43. 10.1021/acsami.1c11775 34428887

[103] Chen Y-J, Wen Y-Y, Li W-H et al. TiO2@COF nanowire arrays: a “filter amplifier” heterojunction strategy to reverse the redox nature. Nano Lett 2023; 23: 3614–22. 10.1021/acs.nanolett.3c00804 37017682

[104] Zhang M, Chang J-N, Chen Y et al. Controllable synthesis of COFs-based multicomponent nanocomposites from core-shell to yolk-shell and hollow-sphere structure for artificial photosynthesis. Adv Mater 2021; 33: 2105002. 10.1002/adma.202105002 34561905

[105] Zhang X, Wu X, Chen R et al. A triazine-based covalent organic framework decorated with cadmium sulfide for efficient photocatalytic hydrogen evolution from water. J Colloid Interface Sci 2024; 665: 100–8. 10.1016/j.jcis.2024.03.104 38518422

[106] Guo S, Zhao K, Liang L et al. Fully conjugated sp^2^ carbon-linked covalent organic frameworks enables accelerated exciton process for superior singlet oxygen photosynthesis for water remediation. Angew Chem Int Ed 2025; 64: e202509141. 10.1002/anie.202509141 40418210

[107] Fan H, Peng M, Strauss I et al. MOF-in-COF molecular sieving membrane for selective hydrogen separation. Nat Commun 2021; 12: 38. 10.1038/s41467-020-20298-7 33397939 PMC7782778

[108] Gao M-L, Qi M-H, Liu L et al. An exceptionally stable core-shell MOF/COF bifunctional catalyst for a highly efficient cascade deacetalization-Knoevenagel condensation reaction. Chem Commun 2019; 55: 6377–80. 10.1039/C9CC02174D 31089619

[109] Xia B, Liu G, Fan K et al. Boosting hydrogen peroxide photosynthesis via a 1D/2D S-scheme heterojunction constructed by a covalent triazine framework with dual O_2_ reduction centers. Chin J Catal 2025; 69: 315–26. 10.1016/S1872-2067(24)60210-X

[110] Chang S, Feng Y, Zhao Y et al. Fabrication of *p-n* heterostructured photocatalysts with triazine-based covalent organic framework and CuInS_2_ for high-efficiency CO_2_ reduction. ACS Appl Mater Interfaces 2024; 16: 13839–48. 10.1021/acsami.3c19525 38446719

[111] Chen C, Ma W, Zhao J. Semiconductor-mediated photodegradation of pollutants under visible-light irradiation. Chem Soc Rev 2010; 39: 4206–19. 10.1039/b921692h 20852775

[112] Akbar K, Moretti E, Vomiero A. Carbon dots for photocatalytic degradation of aqueous pollutants: recent advancements. Adv Opt Mater 2021; 9: 2100532. 10.1002/adom.202100532

[113] Monfort O, Plesch G. Bismuth vanadate-based semiconductor photocatalysts: a short critical review on the efficiency and the mechanism of photodegradation of organic pollutants. Environ Sci Pollut Res 2018; 25: 19362–79. 10.1007/s11356-018-2437-9 29860700

[114] Bi R-X, Liu X, Lei L et al. Core-shell MOF@COF photocatalysts for synergistic enhanced U(VI) and tetracycline cleanup through space and carrier separation. Chem Eng J 2024; 485: 150026. 10.1016/j.cej.2024.150026

[115] Khaing KK, Yin D, Ouyang Y et al. Fabrication of 2D-2D heterojunction catalyst with covalent organic framework (COF) and MoS_2_ for highly efficient photocatalytic degradation of organic pollutants. Inorg Chem 2020; 59: 6942–52. 10.1021/acs.inorgchem.0c00422 32379962

[116] Bi J, Zhang Z, Tian J et al. Interface engineering in a nitrogen-rich COF/BiOBr S-scheme heterojunction triggering efficient photocatalytic degradation of tetracycline antibiotics. J Colloid Interface Sci 2024; 661: 761–71. 10.1016/j.jcis.2024.01.213 38325174

[117] Wang J, Yin D, Guo X et al. Fabrication of a covalent organic framework-based heterojunction via coupling with ZnAgInS nanosphere with high photocatalytic activity. Langmuir 2022; 38: 4680–91. 10.1021/acs.langmuir.2c00203 35394281

[118] Zhang H-X, Ma S-H, Wang H-X et al. Covalently linked MOF@COF direct Z-scheme heterojunction for visible light-driven photocatalytic degradation of flotation agents. J Environ Chem Eng 2024; 12: 111899. 10.1016/j.jece.2024.111899

[119] Yang M, Mao Y, Wang B et al. Heterometallic Mg@Fe-MIL-101/TpPa-1-COF grown on stainless steel mesh: enhancing photo-degradation, fluorescent detection and toxicity assessment for tetracycline hydrochloride. Colloids Surf A 2021; 631: 127725. 10.1016/j.colsurfa.2021.127725

[120] Liu J, Feng C, Li Y et al. Photocatalytic detoxification of hazardous pymetrozine pesticide over two-dimensional covalent-organic frameworks coupling with Ag_3_PO_4_ nanospheres. Sep Purif Technol 2022; 288: 120644. 10.1016/j.seppur.2022.120644

[121] Li L, Yin D, Xiandi G. Construction of a novel 2D-2D heterojunction by coupling a covalent organic framework and In_2_S_3_ for photocatalytic removal of organic pollutants with high efficiency. New J Chem 2021; 45: 15789–800. 10.1039/D1NJ03133C

[122] Ge S, Cai Y, Deng L et al. Constructing heptazine-COF@TiO_2_ heterojunction photocatalysts for efficient photodegradation of acetaminophen under visible light. Chempluschem 2024; 89: e202400139. 10.1002/cplu.202400139 38470161

[123] Zhao Y, Zhang Y, Wang L et al. Efficient H_2_O_2_ production coupling Rhodamine B degradation over covalent organic framework/g-C_3_N_4_ with S-scheme charge separation mechanism and fully hole-electron utilization ability. J Mater Sci Technol 2025; 229: 213–22. 10.1016/j.jmst.2024.12.040

[124] Lin J, Pan H, Chen Z et al. Graphene-based nanomaterials for solar-driven overall water splitting. Chem Euro J 2022; 28: e202200722. 10.1002/chem.202200722 35417051

[125] Zhao H, Yuan Z. Progress and perspectives for solar-driven water electrolysis to produce green hydrogen. Adv Energy Mater 2023; 13: 2300254. 10.1002/aenm.202300254

[126] Xiang C, Weber AZ, Ardo S et al. Modeling, simulation, and implementation of solar-driven water-splitting devices. Angew Chem Int Ed 2016; 55: 12974–88. 10.1002/anie.201510463 27460923

[127] Chen S, Thind SS, Chen A. Nanostructured materials for water splitting-state of the art and future needs: a mini-review. Electrochem Commun 2016; 63: 10–7. 10.1016/j.elecom.2015.12.003

[128] Navalon S, Dhakshinamoorthy A, Alvaro M et al. Metal-organic frameworks as photocatalysts for solar-driven overall water splitting. Chem Rev 2023; 123: 445–90. 10.1021/acs.chemrev.2c00460 36503233 PMC9837824

[129] Ding Q, Song B, Xu P et al. Efficient electrocatalytic and photoelectrochemical hydrogen generation using MoS_2_ and related compounds. Chem 2016; 1: 699–726. 10.1016/j.chempr.2016.10.007

[130] Tachibana Y, Vayssieres L, Durrant JR. Artificial photosynthesis for solar water-splitting. Nature Photon 2012; 6: 511–8. 10.1038/nphoton.2012.175

[131] Fujishima A, Honda K. Electrochemical photolysis of water at a semiconductor electrode. Nature 1972; 238: 37–8. 10.1038/238037a0 12635268

[132] Chen W, Xue P, Wang Z et al. A porous polyacrylonitrile (PAN)/covalent organic framework (COF) fibrous membrane photocatalyst for highly efficient and ultra-stable hydrogen evolution. J Colloid Interface Sci 2023; 652: 341–9. 10.1016/j.jcis.2023.08.039 37597415

[133] Cheng J, Wu Y, Zhang W et al. Fully conjugated 2D sp^2^ carbon-linked covalent organic frameworks for photocatalytic overall water splitting. Adv Mater 2024; 36: 2305313. 10.1002/adma.202305313 37818737

[134] Shen R, Liang G, Hao L et al. In situ synthesis of chemically bonded 2D/2D covalent organic frameworks/O-vacancy WO_3_ Z-scheme heterostructure for photocatalytic overall water splitting. Adv Mater 2023; 35: 2303649. 10.1002/adma.202303649 37319036

[135] Chen Y-J, Zhang J-Z, Wu Z-X et al. Molecular engineering of perylene diimide polymers with a robust built-in electric field for enhanced solar-driven water splitting. Angew Chem Int Ed 2024; 63: e202318224. 10.1002/anie.202318224 38095880

[136] Chong W-K, Ng B-J, Lee YJ et al. Self-activated superhydrophilic green ZnIn_2_S_4_ realizing solar-driven overall water splitting: close-to-unity stability for a full daytime. Nat Commun 2023; 14: 7676. 10.1038/s41467-023-43331-x 37996415 PMC10667227

[137] Wan S, Wang W, Cheng B et al. A superlattice interface and S-scheme heterojunction for ultrafast charge separation and transfer in photocatalytic H_2_ evolution. Nat Commun 2024; 15: 9612. 10.1038/s41467-024-53951-6 39511168 PMC11543929

[138] Wan S, Xu J, Cao S et al. Promoting intramolecular charge transfer of graphitic carbon nitride by donor-acceptor modulation for visible-light photocatalytic H_2_ evolution. Interdiscip Mater 2022; 1: 294–308.

[139] Qiao F . Photoelectrocatalytic hydrogen production: hydrogen production principle, performance optimization strategy, application and prospect. Nano Res Energy 2025; 4: e9120132. 10.26599/NRE.2024.9120132

[140] Liu Y, Jiang L, Tian Y et al. Covalent organic framework/g-C_3_N_4_ van der Waals heterojunction toward H_2_ production. Inorg Chem 2023; 62: 3271–7. 10.1021/acs.inorgchem.2c04366 36755483

[141] Thote J, Aiyappa HB, Deshpande A et al. A covalent organic framework-cadmium sulfide hybrid as a prototype photocatalyst for visible-light-driven hydrogen production. Chem Euro J 2014; 20: 15961–5. 10.1002/chem.201403800 25307944

[142] Wang Y, Hu Z, Wang W et al. Design of well-defined shell-core covalent organic frameworks/metal sulfide as an efficient Z-scheme heterojunction for photocatalytic water splitting. Chem Sci 2021; 12: 16065–73. 10.1039/D1SC05893B 35024128 PMC8672765

[143] Wang T, Yang B, Zhou Z et al. Designing organic-inorganic semiconductor heterojunctions based on COFs for efficient photocatalytic hydrogen evolution. Small 2025; 21: e2501128. 10.1002/smll.202501128 40130777

[144] Zhang H-Y, Yang Y, Li C-C et al. A new strategy for constructing covalently connected MOF@COF core-shell heterostructures for enhanced photocatalytic hydrogen evolution. J Mater Chem A 2021; 9: 16743–50. 10.1039/D1TA04493A

[145] Chen C, Xiong Y, Zhong X et al. Enhancing photocatalytic hydrogen production via the construction of robust multivariate Ti-MOF/COF composites. Angew Chem Int Ed 2022; 61: e202114071. 10.1002/anie.202114071 34780112

[146] Zhang F-M, Sheng J-L, Yang Z-D et al. Rational design of MOF/COF hybrid materials for photocatalytic H_2_ evolution in the presence of sacrificial electron donors. Angew Chem Int Ed 2018; 57: 12106–10. 10.1002/anie.201806862 30022581

[147] Xu M, Lu M, Qin G et al. Piezo-photocatalytic synergy in BiFeO_3_@COF Z-scheme heterostructures for high-efficiency overall water splitting. Angew Chem Int Ed 2022; 61: e202210700. 10.1002/anie.202210700 36098495

[148] Yan M, Jiang F, Wu Y. Metal-free 2D-2D black phosphorus/covalent organic framework p-n heterojunction for efficient visible-light-driven hydrogen evolution without cocatalysts. Int J Hydrogen Energy 2023; 48: 8867–76. 10.1016/j.ijhydene.2022.12.002

[149] Sun L, Wang W, Kong T et al. Fast charge transfer kinetics in an inorganic-organic S-scheme heterojunction photocatalyst for cooperative hydrogen evolution and furfuryl alcohol upgrading. J Mater Chem A 2022; 10: 22531–9. 10.1039/D2TA06468E

[150] Si W, Yang J, Cao Y et al. Construction of rGO/BP/COF high-low heterojunction for efficient photocatalytic hydrogen evolution. J Alloys Compd 2023; 968: 172218. 10.1016/j.jallcom.2023.172218

[151] Sun G, Zhang J, Cheng B et al. Bifunctional CdS/COF S-scheme photocatalyst for enhanced H_2_ evolution and organic synthesis. Chem Eng J 2023; 476: 146818. 10.1016/j.cej.2023.146818

[152] Dong P, Zhang A, Cheng T et al. 2D/2D S-scheme heterojunction with a covalent organic framework and g-C_3_N_4_ nanosheets for highly efficient photocatalytic H_2_ evolution. Chin J Catal 2022; 43: 2592–605. 10.1016/S1872-2067(22)64094-4

[153] Yi J, Zhang L, Wang W et al. Constructing the covalent organic framework and In_2_O_3_ composites via covalent bonds towards excellent visible-light photocatalytic hydrogen evolution. Fuel 2024; 355: 129470. 10.1016/j.fuel.2023.129470

[154] Yang HZ, Wan YQ, Cheng QR et al. Constructing novel hyper-crosslinked In_2_S_3_@HLZU-1 through molecular expansion for enhanced photocatalytic performance. Environ Sci: Nano 2022; 9: 4268–82. 10.1039/D2EN00676F

[155] Han W, Shao L, Sun X et al. Constructing Cu ion sites in MOF/COF heterostructure for noble-metal-free photoredox catalysis. Appl Catal B 2022; 317: 121710. 10.1016/j.apcatb.2022.121710

[156] Ren JL, Xia ZL, Luo BF et al. Fabrication of 2D/2D COF/SnNb_2_O_6_ nanosheets and their enhanced solar hydrogen production. Inorg Chem Front 2021; 8: 1686–94. 10.1039/D0QI01443E

[157] Shen H, Shang D, Li L et al. Rational design of 2D/2D covalent-organic framework/TiO_2_ nanosheet heterojunction with boosted photocatalytic H_2_ evolution. Appl Surf Sci 2022; 578: 152024. 10.1016/j.apsusc.2021.152024

[158] Zhang Y, Han W, Yang Y et al. S-scheme heterojunction of black TiO_2_ and covalent-organic framework for enhanced evolution. Chem Eng J 2022; 446: 137213. 10.1016/j.cej.2022.137213

[159] Xue R, Zhang R, Liu YS et al. In situ growth of highly dispersed TiO_2_ in covalent organic frameworks for photocatalytic H_2_ evolution. Inorg Chem 2025; 64: 8074–81. 10.1021/acs.inorgchem.5c00134 40088158

[160] Zhao X, Lei M, Ma X et al. Improved photocatalytic hydrogen production with the π-d conjugation between amino groups in COFs and CoS_2_, along with the S-scheme heterojunction. J Catal 2025; 446: 116086. 10.1016/j.jcat.2025.116086

[161] Liu Z, Zhang Y, Wu Y et al. In situ XPS evidence of fully conjugated COF and C_3_N_4_ construct S-scheme heterojunction boosting photogenerated carriers transfer and separate for efficiently photocatalytic hydrogen evolution. J Mater Sci Technol 2025; 233: 48–57. 10.1016/j.jmst.2025.01.040

[162] Wan L, Chen R, Cheung DWF et al. Solar driven CO_2_ reduction: from materials to devices. J Mater Chem A 2023; 11: 12499–520. 10.1039/D3TA00267E

[163] Zhang T, Han X, Nguyen NT et al. TiO_2_-based photocatalysts for CO_2_ reduction and solar fuel generation. Chin J Catal 2022; 43: 2500–29. 10.1016/S1872-2067(21)64045-7

[164] Wang J, Lin S, Tian N et al. Nanostructured metal sulfides: classification, modification strategy, and solar-driven CO_2_ reduction application. Adv Funct Mater 2021; 31: 2008008. 10.1002/adfm.202008008

[165] Zhao Y, Waterhouse GIN, Chen G et al. Two-dimensional-related catalytic materials for solar-driven conversion of CO*_x_* into valuable chemical feedstocks. Chem Soc Rev 2019; 48: 1972–2010. 10.1039/C8CS00607E 30357195

[166] Li Q, Chang J-N, Wang Z et al. Modulated connection modes of redox units in molecular junction covalent organic frameworks for artificial photosynthetic overall reaction. J Am Chem Soc 2023; 145: 23167–75. 10.1021/jacs.3c07471 37820308

[167] Shi X, Huang Y, Long R et al. Sustainable all-weather CO_2_ utilization by mimicking natural photosynthesis in a single material. Natl Sci Rev 2024; 11: nwad275. 10.1093/nsr/nwad275 38226176 PMC10789249

[168] Yin H-Q, Zhang Z-M, Lu T-B. Ordered integration and heterogenization of catalysts and photosensitizers in metal-/covalent-organic frameworks for boosting CO_2_ photoreduction. Acc Chem Res 2023; 56: 2676–87. 10.1021/acs.accounts.3c00380 37707286

[169] Zhang M, Lu M, Lang Z-L et al. Semiconductor/covalent-organic-framework Z-scheme heterojunctions for artificial photosynthesis. Angew Chem Int Ed 2020; 59: 6500–6. 10.1002/anie.202000929 31989745

[170] Rangappa AP, Kumar DP, Do KH et al. Synthesis of pore-wall-modified stable COF/TiO_2_ heterostructures via site-specific nucleation for an enhanced photoreduction of carbon dioxide. Adv Sci 2023; 10: 2300073. 10.1002/advs.202300073 PMC10190585 36965101

[171] Cheng J, Wang W, Zhang J et al. Confining quantum dots within covalent organic framework cages for coupled CO_2_ photoreduction and value-added chemical synthesis. Adv Mater 2025; 37: e12144. 10.1002/adma.202512144 40708390 PMC12531737

[172] Huerta-Zeron HD, Rockstroh N, Lang M et al. Photocatalytic CO_2_ reduction with a TiO_2_-supported copper photosensitizer and an iron-based CO_2_ reduction catalyst. Catal Sci Technol 2023; 13: 3940–5. 10.1039/D3CY00572K

[173] Quach T-A, Gopalakrishnan VN, Becerra J et al. Z-scheme heterojunction of chemically integrated COF-366-Co/UiO-66-NH_2_ MOFs nanocomposites for selective production of CO via CO_2_ solar-drive photoreduction. Catal Today 2023; 421: 114218. 10.1016/j.cattod.2023.114218

[174] Wang J, Dai Z, Wang L et al. A Z-scheme heterojunction of porphyrin-based core-shell Zr-MOF@Pro-COF-Br hybrid materials for efficient visible-light-driven CO_2_ reduction. J Mater Chem A 2023; 11: 2023–30. 10.1039/D2TA08333G

[175] Li H, Cheng C, Yang Z et al. Encapsulated CdSe/CdS nanorods in double-shelled porous nanocomposites for efficient photocatalytic CO_2_ reduction. Nat Commun 2022; 13: 6466. 10.1038/s41467-022-34263-z 36309504 PMC9617972

[176] Wang J, Wang L, Zhang D et al. Covalently connected core-shell NH_2_-MIL-125@COFs-OH hybrid materials for visible-light-driven CO_2_ reduction. J Colloid Interface Sci 2023; 637: 1–9. 10.1016/j.jcis.2022.12.154 36682113

[177] Mao J, Wang L, Qu S et al. Defect engineering in CuS_x_/COF hybridized heterostructures: synergistic facilitation of the charge migration for an efficacious photocatalytic conversion of CO_2_ into CO. Inorg Chem 2022; 61: 20064–72. 10.1021/acs.inorgchem.2c03481 36449266

[178] Wang Y, Dai Z, Wang J et al. Scheme-II heterojunction of Bi_2_WO_6_@Br-COFs hybrid materials for CO_2_ photocatalytic reduction. Chem Eng J 2023; 471: 144559. 10.1016/j.cej.2023.144559

[179] Wang Y, Cao Y, Wei S et al. N-Bi covalently connected Z-scheme heterojunction by in situ anchoring BiOCl on triazine-based bromine-substituted covalent organic frameworks for the enhanced photocatalytic reduction of CO_2_ and Cr (VI). Chem Eng J 2025; 505: 159349. 10.1016/j.cej.2025.159349

[180] Yang J, Zeng X, Tebyetekerwa M et al. Engineering 2D photocatalysts for solar hydrogen peroxide production. Adv Energy Mater 2024; 14: 2400740. 10.1002/aenm.202400740

[181] Hou H, Zeng X, Zhang X. Production of hydrogen peroxide by photocatalytic processes. Angew Chem Int Ed 2020; 59: 17356–76. 10.1002/anie.201911609 31571331

[182] Zeng X, Liu Y, Hu X et al. Photoredox catalysis over semiconductors for light-driven hydrogen peroxide production. Green Chem 2021; 23: 1466–94. 10.1039/D0GC04236F

[183] Zhang M, Huang P, Li R-H et al. Reticular synthesis of 3D metal cluster-based COFs with record high-connectivity for efficient photocatalytic H_2_O_2_ synthesis. Angew Chem Int Ed 2025; 64: e202507624. 10.1002/anie.202507624 40461438

[184] Zhang N, Lin S, Wang F et al. Highly efficient photocatalytic H_2_O_2_ production on core-shell CdS@CdIn_2_Sb_4_ heterojunction in non-sacrificial system. Res Chem Intermed 2021; 47: 3379–93. 10.1007/s11164-021-04467-x

[185] Mehrotra R, Oh D, Jang J. Unassisted selective solar hydrogen peroxide production by an oxidised buckypaper-integrated perovskite photocathode. Nat Commun 2021; 12: 6644. 10.1038/s41467-021-26832-5 34789721 PMC8599672

[186] Shi H, Li Y, Wang X et al. Selective modification of ultra-thin g-C_3_N_4_ nanosheets on the (110) facet of Au/BiVO_4_ for boosting photocatalytic H_2_O_2_ production. Appl Catal B 2021; 297: 120414. 10.1016/j.apcatb.2021.120414

[187] Cheng J, Wang W, Zhang J et al. Molecularly tunable heterostructured co-polymers containing electron-deficient and -rich moieties for visible-light and sacrificial-agent-free H_2_O_2_ photosynthesis. Angew Chem Int Ed 2024; 63: e202406310. 10.1002/anie.202406310 38712550

[188] Chen Z, Wan S, Cheng B et al. Efficient overall photosynthesis of H_2_O_2_ by the BTz@Mn_0.2_Cd_0.8_S S-scheme heterojunction. Sci China Chem 2024; 67: 1953–60. 10.1007/s11426-024-2012-5

[189] Ma T, Huang G, Wang X et al. Photochromic radical states in 3D covalent organic frameworks with zyg topology for enhanced photocatalysis. Natl Sci Rev 2024; 11: nwae177. 10.1093/nsr/nwae177 38883289 PMC11173181

[190] Yap FM, Sheng Ling GZ, Su BJ et al. Recent advances in structural modification on graphitic carbon nitride (g-C_3_N_4_)-based photocatalysts for high-efficiency photocatalytic H_2_O_2_ production. Nano Res Energy 2024; 3: e9120091. 10.26599/NRE.2023.9120091

[191] Chen H, Gao S, Huang G et al. Built-in electric field mediated S-scheme charge migration in COF/In_2_S_3_ heterojunction for boosting H_2_O_2_ photosynthesis and sterilization. Appl Catal B 2024; 343: 123545. 10.1016/j.apcatb.2023.123545

[192] Gu M, Yang Y, Cheng B et al. Unveiling product selectivity in S-scheme heterojunctions: harnessing charge separation for tailored photocatalytic oxidation. Chin J Catal 2024; 59: 185–94. 10.1016/S1872-2067(23)64610-8

[193] Jiang Z, Zhang J, Cheng B et al. Hollow TiO_2_@TpPa S-scheme photocatalyst for efficient H_2_O_2_ production through ^1^O_2_ in deionized water using phototautomerization. Small 2025; 21: e2409079. 10.1002/smll.202409079 39865987

[194] Qi M-Y, Conte M, Anpo M et al. Cooperative coupling of oxidative organic synthesis and hydrogen production over semiconductor-based photocatalysts. Chem Rev 2021; 121: 13051–85. 10.1021/acs.chemrev.1c00197 34378934

[195] Das S, Perez-Ramirez J, Gong J et al. Core-shell structured catalysts for thermocatalytic, photocatalytic, and electrocatalytic conversion of CO_2_. Chem Soc Rev 2020; 49: 2937–3004. 10.1039/C9CS00713J 32407432

[196] Wang H, Wang H, Wang Z et al. Covalent organic framework photocatalysts: structures and applications. Chem Soc Rev 2020; 49: 4135–65. 10.1039/D0CS00278J 32421139

[197] Peng YZ, Guo GC, Guo S et al. Charge transfer from donor to acceptor in conjugated microporous polymer for enhanced photosensitization. Angew Chem Int Ed 2021; 60: 22062–9. 10.1002/anie.202109968 34342372

[198] Wang K, Li C, Zhang G et al. Donor-acceptor engineering of a triplet-exciton-optimized MOF photocatalyst for efficient singlet oxygen-mediated oxidation. Natl Sci Rev 2025; 12: nwaf024. 10.1093/nsr/nwaf024 40060922 PMC11887853

[199] Liu X, Bi R-X, Zhang C-R et al. SnS_2_-covalent organic framework Z-scheme van der Waals heterojunction for enhanced photocatalytic reduction of uranium (VI) in rare earth tailings wastewater. Chem Eng J 2023; 460: 141756. 10.1016/j.cej.2023.141756

[200] Feng C, Hu J, Xiao C et al. Shell-core COF@Co_3_O_4_ Z-scheme heterojunctions for triple amplification of oxidative stress to enhance nanocatalytic-sonodynamic tumor therapy. Chem Eng J 2023; 460: 141874. 10.1016/j.cej.2023.141874

[201] Bao T, Xi Y, Zhang C et al. Highly efficient nitrogen fixation over S-scheme heterojunction photocatalysts with enhanced active hydrogen supply. Natl Sci Rev 2024; 11: nwae093. 10.1093/nsr/nwae093 38577667 PMC10989659

[202] Zeng J-Y, Wang X-S, Liu X-H et al. Light-driven biohybrid system utilizes N_2_ for photochemical CO_2_ reduction. Natl Sci Rev 2023; 10: nwad142. 10.1093/nsr/nwad142 37426486 PMC10325001

